# Phytohormones in a universe of regulatory metabolites: lessons from jasmonate

**DOI:** 10.1093/plphys/kiae045

**Published:** 2024-01-30

**Authors:** Debora Gasperini, Gregg A Howe

**Affiliations:** Department of Molecular Signal Processing, Leibniz Institute of Plant Biochemistry, Halle 06120, Germany; Department of Energy-Plant Research Laboratory, Michigan State University, East Lansing, MI 48824, USA; Department of Biochemistry and Molecular Biology, Michigan State University, East Lansing, MI 48824, USA; Plant Resilience Institute, Michigan State University, East Lansing, MI 42284, USA

## Abstract

Small-molecule phytohormones exert control over plant growth, development, and stress responses by coordinating the patterns of gene expression within and between cells. Increasing evidence indicates that currently recognized plant hormones are part of a larger group of regulatory metabolites that have acquired signaling properties during the evolution of land plants. This rich assortment of chemical signals reflects the tremendous diversity of plant secondary metabolism, which offers evolutionary solutions to the daunting challenges of sessility and other unique aspects of plant biology. A major gap in our current understanding of plant regulatory metabolites is the lack of insight into the direct targets of these compounds. Here, we illustrate the blurred distinction between classical phytohormones and other bioactive metabolites by highlighting the major scientific advances that transformed the view of jasmonate from an interesting floral scent to a potent transcriptional regulator. Lessons from jasmonate research generally apply to other phytohormones and thus may help provide a broad understanding of regulatory metabolite–protein interactions. In providing a framework that links small-molecule diversity to transcriptional plasticity, we hope to stimulate future research to explore the evolution, functions, and mechanisms of perception of a broad range of plant regulatory metabolites.

## Teaser

Lessons from research on jasmonate and other small-molecule phytohormones inform efforts to understand how secondary metabolites acquire signaling roles and mediate transcriptional flexibility.

**ADVANCES**

- The past century of plant hormone research has helped reveal how low-molecular-weight metabolites modulate transcription to promote plasticity in growth, development, and environmental responses.
- Recent knowledge of the evolutionary origins of phytohormone biosynthesis, perception, and signaling is helping redefine the hormone concept to reflect the unique biology of plants.
- Currently recognized phytohormones are part of a larger group of metabolites that alter gene expression with different biological effects and degrees of conservation within land plants.
- Classical genetic approaches to study jasmonates and other phytohormones provide a roadmap to exploring the activities of other regulatory metabolites.
- Advances in mass spectrometry, molecular modeling, and artificial intelligence-based structural prediction provide new tools to identify regulatory metabolite–protein interactions in plants.

## Introduction

Plants use a diverse repertoire of chemical messengers to coordinate their growth and development with informational cues from the environment. Among organic signaling compounds, small-molecule plant hormones (i.e. phytohormones) have been the focus of much research during the past century. Currently recognized phytohormones influence virtually all aspects of growth, development, and environmental acclimation and tend to be broadly conserved among land plants. Evolutionary reconstruction of plant hormone response pathways using phylogenomic approaches has provided important insights into the emergence of small molecules as drivers of phenotypic plasticity and innovations in life history traits (Bowman et al. 2017; Blázquez et al. 2020). Moreover, the molecular mechanisms governing phytohormone homeostasis and action have emerged as key targets for improving the productivity and stress resilience of crops (Hedden 2003; Bailey-Serres et al. 2019).

What is the meaning of “hormone” as it applies to the unique biology of plants? Beyond the limited number of compounds elevated to phytohormone status in textbooks, what other plant metabolites have signaling properties? What are the mechanisms by which these molecules are perceived and transduced to downstream effectors? How do such metabolites modulate transcription, and how are these functions acquired during evolution? Finally, how can this knowledge inform the development of new crop varieties that are more productive, nutritious, and better adapted to changing climate conditions? These are the main questions that motivated this topical review.

## Phytohormones as regulatory metabolites

Here, we develop the hypothesis that currently recognized plant hormones are part of a larger and more diverse group of metabolites that acquired regulatory attributes over the course of embryophyte evolution. This supposition is based on the increasing recognition that many secondary (also referred to as “specialized”) metabolites exhibit hormone-like effects on growth, development, and responses to the environment. The mechanisms by which these bioactive compounds act remain largely unknown but are often associated with changes in gene expression. The past century of plant hormone research, combined with new approaches to systematically map protein–metabolite interactions, provides a general roadmap for future progress in this area. We hope that this conceptual framework helps guide future research aimed at understanding the evolution, functions, and mechanisms of action of plant regulatory metabolites beyond currently recognized phytohormones.

### Overview of phytohormone actions and origins

The identification of genes required for plant hormone biosynthesis and action resulted largely from the use of genetic approaches applied to the model eudicot species *Arabidopsis* (*Arabidopsis thaliana*). This branch of research greatly advanced our understanding of how “linear” phytohormone-response pathways function at the molecular level, including the discovery of ethylene as the first gaseous signaling molecule (Kende 1998), the elucidation of mechanisms to perceive steroids and auxins at the cell surface (He et al. 2000; Yu et al. 2023), and the paradigm that some hormones act as molecular glue to promote the reorganization of transcription complexes (Dharmasiri et al. 2003; Tan et al. 2007; Sheard et al. 2010). A deep mechanistic understanding of phytohormone-response pathways has also provided a foundation on which to explore more complex questions, such as the evolutionary origins of chemical communication in plants, cross-regulation between phytohormone-response pathways, the ecological relevance of phytohormones, and strategies to engineer economically important plant traits.

**Figure 1.**
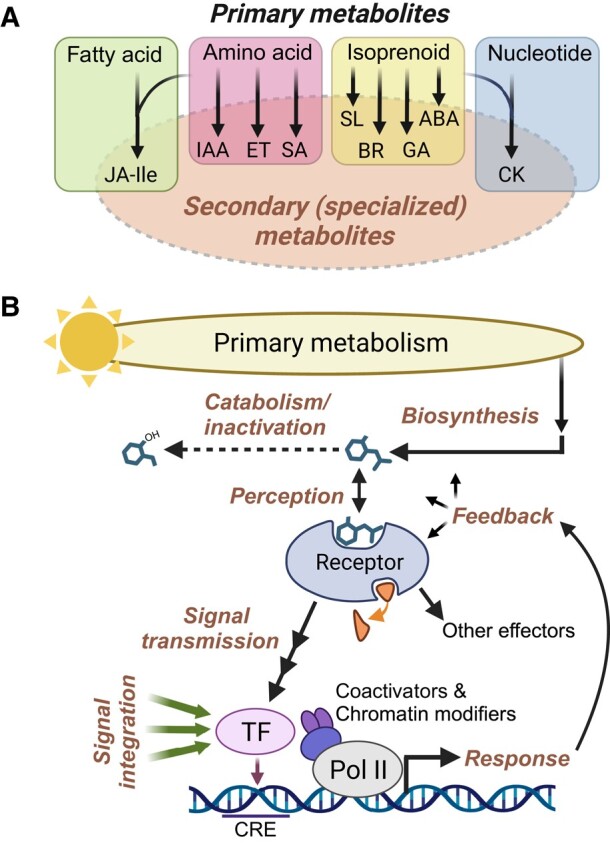
Common themes in plant hormone biosynthesis and signaling. **A)** Phytohormones can be viewed as secondary (i.e. specialized) metabolites that are derived from several major classes of primary metabolites. Some hormones, including JA-Ile and cytokinin (CK), are synthesized from more than one primary metabolite class. Peptide hormones are omitted for simplicity. **B)** Phytohormone-response pathways consist of several highly integrated processes (bold text) that collectively modulate the activity of one or more effectors. Hormone biosynthetic pathways utilize precursors from primary metabolism and give rise to bioactive compounds whose intracellular concentrations are several orders of magnitude below those of primary metabolic precursors. Bioactive hormones (depicted as a generic chemical structure) are subject to oxidation, conjugation, and other catabolic reactions that inactivate the signal (dashed arrow). Hormone perception is mediated by specific proteinaceous receptors. Many receptors are associated with a ligand-induced enzymatic activity (small curved arrow). Ligand binding transmits the signal to a downstream effector such as a TF. Effector TFs bind to specific *cis*-regulatory elements (CRE) in response genes and work together with various coactivators and chromatin-modifying enzymes to control the rate of transcription via RNA polymerase II (Pol II). TFs and other effectors generate physiological responses. These responses are subject to multiple levels of feedback regulation, for example, as a means of preventing the overactivation of a response. Hormone-responsive TFs are subject to many additional layers of regulation, including inputs from multiple developmental and environmental signals (green arrows). As a consequence, the expression of any given target gene is context dependent; effector TFs constitute the apex of the regulatory hierarchy, with hormone-receptor inputs serving a condition-specific role. ABA, abscisic acid; BR, brassinosteroid; ET, ethylene; GA, gibberellin; IAA, indole acetic acid (auxin); SA, salicylic acid; SL, strigolactone.

Currently recognized plant hormones exert their effects by triggering signal transduction pathways that modulate the activities of specific cellular effectors, most often transcription factors (TFs). It is now evident that the phenotypic outputs of hormone signaling cascades are strongly influenced by synergistic and antagonistic interactions with many other signaling pathways (Bari and Jones 2009; Santner et al. 2009; Altmann et al. 2020). Due to this interconnectivity, phytohormone-response networks regulate gene expression and other physiological processes in a context-specific manner that is shaped by multiple developmental and environmental (e.g. light) cues (Fig. 1). This complex web of interactions likely explains the pleiotropic effects of hormones and why they appear to “do” so many things. A better understanding of how phytohormones and other bioactive metabolites interact to influence gene expression networks has the potential to improve crop performance and thus represents a major goal of modern plant science (Berens et al. 2017).

Plant hormones are derived from nearly all classes of primary metabolites, including isoprenoids, amino acids, fatty acids, and nucleotides (Fig. 1 and Box 1). Given their potent and wide-ranging effects on gene expression, endogenous hormone concentrations are subject to exquisite feedback control at the level of biosynthesis, transport, and catabolism. Bioactive end products of hormone biosynthetic pathways are perceived by specific proteinaceous receptors that, upon ligand binding, transduce the signal to a major TF or other effector (Fig. 1). High-affinity binding of hormones to their cognate receptor accounts for the ability of these metabolites to exert physiological effects at very low concentrations. Elucidation of the 3-dimensional structures of hormone–receptor complexes has yielded unprecedented insight into how ligand binding is linked to rapid changes in effector activity (Takeuchi et al. 2021). Some hormones induce a conformational change (i.e. allosteric regulation) in their receptor, whereas others act as molecular glues to promote protein–protein interactions that transduce the signal to an effector. Ligand–receptor interactions typically activate target effectors through changes in the activity of a receptor-linked enzyme, such as a kinase, phosphatase, or ubiquitin ligase. The integration of hormone perception with enzymatic function is further illustrated by the recent discovery of plant hormone receptors having α/β-hydrolase or nucleotide cyclase activity (Takeuchi et al. 2021; Qi et al. 2022).

Box 1. **Plant hormones are found in unexpected places**

**Figure 2.**
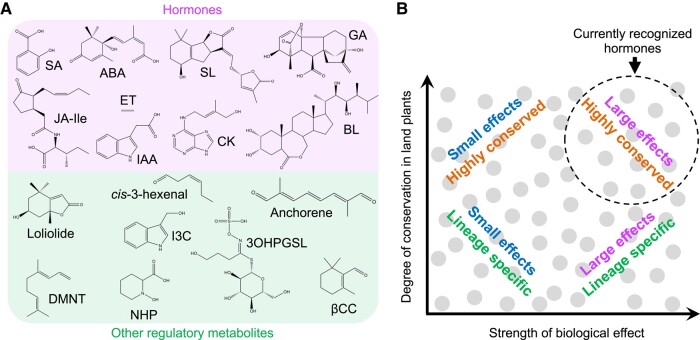
Diversity of plant regulatory metabolites. **A)** Chemical structures of currently recognized phytohormones (top group) and representative regulatory metabolites (bottom group). **B)** Plant regulatory metabolites (gray circles) differ not only in their chemical structures but also in their phylogenetic distribution (*y* axis) and range of biological effects (*x* axis). For example, phytohormone action is typically linked to the differential expression of hundreds (or thousands) of genes, whereas other regulatory metabolites modulate the expression of a smaller set of genes. Owing to their strong biological effects and high degree of conservation in land plants, currently recognized phytohormones tend to occupy the upper right quadrant (dashed circle) of the plot. 3OHPGSL, 3-hydroxypropylglucosinolate; ABA, abscisic acid; BL, brassinolide; CK, cytokinin (*trans*-zeatin); DMNT, (*E*)-3,8-dimethyl-1,4,7-nonatriene; ET, ethylene; GA, gibberellic acid (GA_3_); I3C, indole-3-carbinol; IAA, indole-3-acetic acid (auxin); Jasmonic acid (JA)-Ile, jasmonoyl-L-isoleucine; NHP, *N*-hydroxy-pipecolic acid; SA, salicylic acid; SL, strigolactone (strigol); βCC, β-cyclocitral.

The tendency of phytohormones to exert broad-spectrum effects (Fig. 2) may reflect their discovery through the use of bioassays designed to identify substances that modulate plant growth and cell division. In this context, it is interesting to note that activity-driven purification of phytohormones employed starting materials not only from plant sources but also complex chemical mixtures from non-plant sources, including coal gas (ethylene), herring sperm DNA (cytokinin), human urine (auxin), and fungal exudates (gibberellins and jasmonates); only later was it determined that these metabolites are endogenous plant compounds. The powerful biological effects of classical hormones may explain why these low-hanging fruits on the larger tree of regulatory metabolites were the first to be identified using bioassay-based approaches biased toward easily measurable phenotypes (e.g. growth) that involve large-scale reprogramming of gene expression. Endogenous regulatory metabolites with more subtle effects on a narrow range of effectors surely exist (Fig. 2), but alternative experimental approaches may be needed to identify them (see Concluding remarks and future perspectives). Many phytohormones are produced by nonplant taxa, including bacteria, fungi, and algae, which lack the canonical hormone response pathways found in land plants. The production of phytohormones in these lineages has fascinating implications for understanding how processes such as horizontal gene transfer and endosymbiosis have shaped the relationships of plants with other groups of organisms (Bari and Jones 2009; Morffy and Strader 2020).

The advent of functional phylogenomic approaches has spurred remarkable progress in understanding the evolution of phytohormone-response pathways and, more broadly, the understudied interface between metabolism and signaling (Bowman et al. 2017; Blázquez et al. 2020; Rieseberg et al. 2023). Small-molecule plant hormones have features ascribed to both primary and secondary metabolites (Erb and Kliebenstein 2020; Fàbregas and Fernie 2021). Primary metabolites, such as amino acids, fatty acids, and sugars, are relatively abundant compounds (e.g. micromolar range) whose critical roles in the plant lifecycle stem from their use as building blocks that fuel tissue growth and increases in biomass. Although many hormones are also essential for the plant lifecycle, this dependency reflects the signaling properties of these metabolites, which typically accumulate to minuscule levels (e.g. subnanomolar) relative to the primary compounds from which they are derived. Whereas primary metabolites are produced by all organisms and cell types, the distribution of phytohormones in the tree of life is more sporadic and includes recent innovations within land plants (Box 1) (Bowman et al. 2017; Weng et al. 2021; Chini et al. 2023; Rieseberg et al. 2023). These collective observations support the view that small-molecule phytohormones trace their evolutionary origins to specialized metabolites that were co-opted for signaling roles in either the ancestral land plant or within land plants (Bowman et al. 2017; Rieseberg et al. 2023). Accordingly, plants use small-molecule signals in ways that are often unique to the plant kingdom (Box 2).

Box 2. **Phytohormone signaling pathways are unique**

The term “hormone” was coined by animal physiologists to convey the notion of chemical messengers that coordinate the growth and metabolic activity in different parts of the body (Tata 2005). This view was founded on observations that mammalian hormones are produced in specific locations, transported through the bloodstream, and generate physiological effects in target tissues. Plant scientists have generally, but not universally (Trewavas 1981; Bennett and Leyser 2014), embraced the hormone paradigm because it accounts for the potent biological effects of endogenous plant growth and immune regulators (Davies 2010). Beyond a general role in mediating intercellular communication, however, fundamental differences between plant and animal biology suggest that extrapolating hormone signaling mechanisms from animals to plants is naive at best, and at worst hinders a full appreciation of how plants use small molecules as informational cues. Indeed, the independent evolution of multicellularity in plants endowed them with signal communication networks that were likely shaped by unique features of the embryophyte lineage: a thick cell wall that restricts cell mobility; an autotrophic, sessile lifestyle enabled by photosynthetic plastids; increased developmental flexibility (i.e. totipotency) of interdependent above- and belowground organs; and an extraordinary diversity of metabolites to help meet the profound challenges of sessility. While both plants and animals rely on transcriptional programming to specify patterns of cell identity and growth, the signaling components responsible for executing these developmental programs in plants and animals are largely nonhomologous (Meyerowitz 2002). Moreover, many of the storied signaling components that helped to establish the animal hormone paradigm, including G protein-coupled receptors and associated second messengers such as cyclic AMP, either appear to be missing in plants (Urano and Jones 2014) or function in plants in ways that deviate from the animal paradigm (Wong et al. 2023). Plant cells also appear to be more autonomous than their animal counterparts, such that most plant cell types likely have the capacity to both synthesize and perceive a given chemical signal. Accordingly, the classic hormone concept of “action at a distance” does not necessarily apply to plants, even though many plant signaling compounds are transported long distances. If we have learned anything from the past century of phytohormone research, it is that plants use low-molecular-weight metabolites as informational cues in ways that are often unique to the plant kingdom.

### Phytohormones versus regulatory metabolites: what's the difference?

There is general consensus about the properties that define a phytohormone (Davies 2010). These characteristics include the ability to pleiotropically modulate growth, developmental, and immune-related processes, activity at very low concentrations, rapid biological responses to fluctuating concentrations of the hormone signal, and a high degree of conservation in the plant kingdom. Plant hormones are also perceived by dedicated receptors that transduce the signal to major effectors of physiological change.

Beyond these general features, how should we think about phytohormones conceptually? As is the case for other complex multicellular organisms, the spatial and temporal action of transcriptional regulators (e.g. TFs) plays a dominant role in executing a genetic blueprint that specifies rudimentary plant phenotypes. Phytohormone networks appear to confer plasticity to these hard-wired processes by providing real-time, environmentally dependent inputs that coordinate gene expression patterns between cell types, tissue types, and organs. This conceptual view of phytohormones as conditional modulators of gene expression has implications for understanding the stepwise assembly of hormone-response pathways during evolution (Bowman et al. 2017; Mutte et al. 2018; Blázquez et al. 2020). Increasing evidence indicates that protein components of various phytohormone signaling cascades predated the recruitment of the hormone into the pathway and thus have the capacity to control output responses independently of the hormone (Mutte et al. 2018; Sun et al. 2019; Bunsick et al. 2021). For example, auxin appears to have been recruited as a signal based on its ability to promote protein–protein interactions between preexisting components of the pathway, thereby enabling efficient control of TF activity (Mutte et al. 2018; Cao et al. 2022). In this scenario, the major drivers of plant phenotype are ancient transcriptional regulators such as TFs, which over evolutionary time acquired the capacity to receive and integrate information from internal (e.g. metabolic) and external (e.g. light) cues (Fig. 1).

A unified phytohormone concept also needs to address the lack of a clear distinction between known hormones and an increasing number of plant metabolites that exhibit regulatory properties (Erb and Kliebenstein 2020). Evolutionary theory predicts that plants possess a diverse array of regulatory metabolites that differ not only in their biosynthetic origin and phylogenetic distribution but also in their range of biological effects (Fig. 2). We propose that hormones should be viewed not as an exclusive group of endogenous mediators but rather as part of a spectrum of compounds that acquired signaling attributes during or after the colonization of land by plants. This framework is consistent with current knowledge of how specialized metabolites in general, and phytohormones in particular, evolved (Bowman et al. 2017; Blázquez et al. 2020; Rieseberg et al. 2023). Phylogenomic analyses suggested that most hormone-response pathways emerged early in the evolution of land plants and, because of their essential roles in the plant lifecycle, were retained in present-day lineages (Bowman et al. 2017). Regulatory metabolites whose distribution is restricted to specific plant taxa were either recruited later in evolution or perhaps discarded from other lineages due to their lack of utility in a particular environment. This evolutionary perspective accounts for the existence of lineage-specific regulatory metabolites (e.g. certain glucosinolate derivatives) that confer subtle—and thus difficult-to-detect—fitness advantages in a given environment (Fig. 2) (Erb and Kliebenstein 2020).

While it is obvious that all small-molecule hormones are regulatory metabolites, it is less clear which of these compounds should be categorized as phytohormones. Dwelling on this ambiguous distinction has limited practical value because the evolutionary tinkering that recruits metabolites into their myriad roles is blind to such classification schemes. One solution to this quandary is to phase out the use of the term “hormone” in favor of a more general term such as regulatory metabolite (Bennett and Leyser 2014). A quick survey of the literature, however, suggests that the plant hormone/phytohormone labels are here to stay. Suffice it to say that currently recognized phytohormone pathways mediate potent and pleiotropic biological effects in a wide range of plant species. Debating whether or not a particular metabolite should be classified as a hormone is less meaningful than determining how that compound functions at a mechanistic level; such insights are needed to obtain a satisfactory understanding of the physiological, ecological, and evolutionary relevance of bioactive metabolites.

### The expanding universe of plant regulatory metabolites

Twenty-five years ago, Kende and Zeevaart (1997) predicted that the list of 5 “classical” plant hormones, namely abscisic acid, auxin, cytokinin, gibberellin, and ethylene, would expand as the functions of other bioactive compounds became recognized. This expectation has materialized with the general acceptance of brassinosteroid, jasmonate, salicylic acid, and strigolactone as the newest members of the group (Santner et al. 2009; Takeuchi et al. 2021). Beyond these known phytohormones, to what extent do plant metabolites have signaling attributes and how were these properties acquired during evolution? One way to address this question is through the study of presumptive regulatory metabolites whose chemical identity is unknown. The KARRIKIN INSENSITIVE 2 receptor, which perceives smoke-derived compounds (i.e. karrikins) that promote seed germination, is thought to perceive an endogenous ligand, but the identity of this compound(s) remains elusive (Waters and Nelson 2023). Another example is the cytochrome P450 KLUH/KLU, which in *Arabidopsis* is involved in the production of a mobile growth-promoting factor that is likely distinct from known phytohormones (Anastasiou et al. 2007).

A second approach to expand our knowledge of plant regulatory metabolites is to identify the molecular targets of endogenous compounds that exhibit signaling properties. The list of such compounds is lengthy and includes peptides, sugars (e.g. trehalose-6-phosphate), nucleotides (e.g. cyclic nucleotides), and amino acids (e.g. Glu). The direct targets of several of these compounds have been identified and studied in detail (Sandoval and Santiago 2020). Below, we highlight a few examples of secondary metabolites whose regulatory properties and modes of action are unknown but are under active investigation. Readers are referred to recent reviews for a more in-depth discussion of bioactive plant compounds originating from secondary metabolism (Erb and Kliebenstein 2020; Durán-Medina et al. 2021; Weng et al. 2021; Cai and Aharoni 2022).

Plant secondary metabolites account for much of the chemical diversity in nature. This large assortment of compounds exerts direct effects on plant-interacting organisms and also promotes adaptation to abiotic stress conditions. Interestingly, an increasing number of specialized metabolites are reported to possess signaling attributes reminiscent of phytohormones (Fig. 2) (Erb and Kliebenstein 2020). Flavonoids and polyamines have long been known for their ability to influence gene expression and other physiological processes (Peer and Murphy 2007; Davies 2010; Pourcel et al. 2013). Glucosinolates comprise a phylogenetically restricted group of defense compounds, some of which exert effects on growth and stress resilience (Kim et al. 2015; Malinovsky et al. 2017; Salehin et al. 2019; Katz et al. 2020) (Fig. 2). *N*-hydroxy-pipecolic acid, β-aminobutyric acid, and indole have received increasing attention for their immunomodulatory properties (Erb et al. 2015; Cai and Aharoni 2022; Shields et al. 2022). Chemically diverse isoprenoids, including volatile terpenoids, have been intensively studied for their signaling roles in growth, defense, and interplant communication (Zuo et al. 2019; Bai et al. 2021; Ninkovic et al. 2021; Hu 2022; Wang and Erb 2022). Oxidative cleavage of carotenoids is also a rich source of metabolites with regulatory functions, beyond the well-characterized abscisic acid and strigolactone hormones (Wang et al. 2021). Examples include the promotion of leaf stress responses by β-cyclocitral (Dinneny et al. 2019) and β-ionone (Felemban et al. 2023), the mediation of plant rhizosphere interactions by zaxinone (Wang, Haider, et al. 2019), and the control of root architecture by anchorene (Jia et al. 2019). Oxygenated fatty acids (i.e. oxylipins) and their derivatives, including green leaf volatiles (Matsui 2006), azelaic acid (Jung et al. 2009), and 12-oxo-phytodienoic acid (OPDA) (Wang et al. 2020), are also recognized as signaling compounds. These examples highlight the regulatory properties of plant secondary metabolites and raise the central question: what is their mode of action?

**Figure 3.**
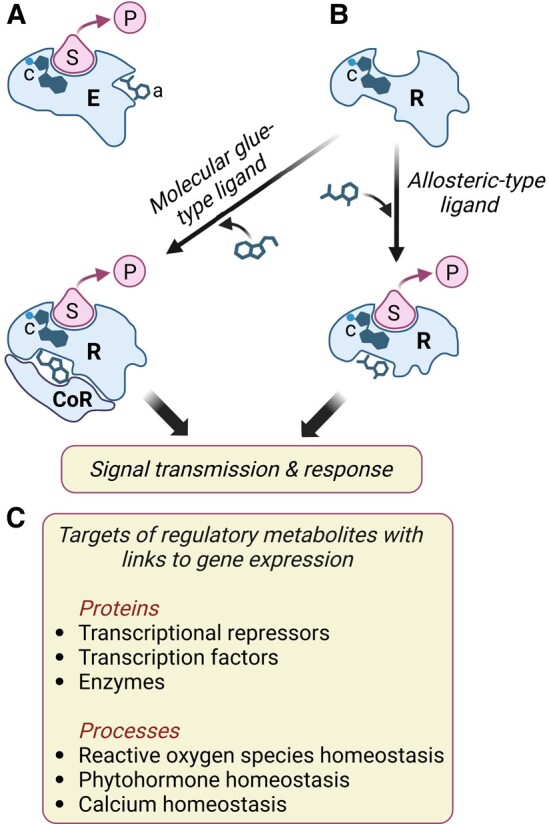
Low-molecular-weight metabolites interact with target proteins to modulate cellular processes that affect gene expression. **A)** Schematic diagram of an enzyme (E) interacting with its substrate (S) to generate a product (P). Enzymes often contain cofactors (c) to assist in catalysis and may also bind allosteric modulators (a) that positively or negatively regulate catalysis through conformational changes in the active site. **B)** Small molecules bind to proteinaceous receptors (R) and transmit information via 2 general mechanisms. First, and analogous to enzyme–metabolite interactions, allosteric-type ligands induce a conformational change in the receptor, which in turn propagates the signal (e.g. ABA, GA, and SL perception). Second, binding of molecular glue-type ligands creates a unique surface that interacts with a coreceptor (CoR) protein (e.g. IAA, JA, and BR perception). Many currently recognized phytohormone receptors possess (or are associated with) an enzymatic activity involved in signal transmission. Some plant receptors, including TIR1 and COI1, are associated with a cofactor (c) that is required for receptor structure and function. **C)** Examples of how gene expression may be modulated by the interactions of specific proteins or cellular processes with low-molecular-weight metabolites other than known phytohormones. See main text for details.

Consistent with their biological activity, most of the above-mentioned bioactive compounds have been shown to modulate nuclear gene expression. Low-molecular-weight metabolites functionally interact with target proteins in well-defined ways, including as enzyme substrates and products, cofactors, allosteric modulators, and molecular glues (Fig. 3). In theory, each of the protein–metabolite interactions has the potential to alter physiological responses through changes in gene expression. Specific examples of how small molecules may regulate transcription include the binding of β-caryophyllene to a TOPLESS-like transcriptional corepressor (Nagashima et al. 2019), the activation of a NIN-like protein TF by nitrate (Liu et al. 2022), the increase in cytosolic Ca^2+^ levels by *Z*-3-hexenal (Aratani et al. 2023), the perception of β-aminobutyric acid by an aspartyl tRNA synthetase (Luna et al. 2014), and the modulation of reactive oxygen species homeostasis by flavonols (Pourcel et al. 2013; Sugimoto et al. 2022; Daryanavard et al. 2023) (Fig. 3). Regulatory metabolites may also alter gene expression networks by affecting the metabolism or action of known phytohormones. For example, auxin signaling and transport are influenced by certain flavonols (Brown et al. 2001; Peer and Murphy 2007), apocarotenoids (Jia et al. 2019), and glucosinolates (Katz et al. 2015, 2020). Priming of plant defense responses by various volatile signals is also mediated by phytohormones, most notably jasmonate and salicylic acid (Fig. 3) (Engelberth et al. 2004; Dani and Loreto 2022; Hu 2022; Li et al. 2022).

It is also likely that the signaling properties of some secondary metabolites reflect dedicated perception systems that remain to be discovered. In their review of secondary metabolites that exhibit signaling properties, Erb and Kliebenstein (2020) concluded that these compounds affect growth and development by mechanisms that “are barely distinguishable from mechanisms normally assigned to plant hormones.” Although this may be true at a conceptual level, studies claiming to identify the direct target of a bioactive plant secondary metabolite are sparse. Additional work is clearly needed to define the physiological relevance and precise modes of action of these and other protein–metabolite interactions.

## Lessons from jasmonate

The past century of plant hormone research, together with emerging technologies to elucidate broader networks of plant metabolite–protein interactions, provides a general road map for future efforts to identify and characterize regulatory metabolites. As a case study, we highlight the major experimental approaches and discoveries that led to the current understanding of jasmonate as a lipid-derived signal for promoting transcriptional flexibility. Detailed mechanistic knowledge of the jasmonate response pathway and its evolutionary origins offer valuable insights into how metabolites are recruited into roles that promote transcriptional pliability in dynamic environments. Although we draw mainly on studies performed with *Arabidopsis* as a model system, increasing attention to other model and nonmodel species has provided key advances into our understanding of jasmonate biology. Readers are referred to several recent reviews for a comprehensive discussion of jasmonate biosynthesis, signaling, and evolution (Chini et al. 2016; Wasternack and Song 2017; Howe et al. 2018; Wasternack and Feussner 2018; Wang, Wu, et al. 2019; Monte 2023).

### Exogenous jasmonates promote diverse responses

The discovery of jasmonates (collectively referred to as JAs) dates back more than half a century to the identification of methyl-JA (MeJA) as a component of floral scent (Demole et al. 1962). This work laid the foundation for the chemical synthesis of JAs, which are widely used as additives in the food and fragrance industries (Wasternack 2015). Jasmonic acid (JA) was first identified as a plant growth inhibitory substance from cultures of the fungus *Lasiodiplodia theobromae* (Aldridge et al. 1971). This finding foreshadowed the current paradigm that pathogens often produce phytohormones or structurally related compounds to co-opt host plant immunity (Box 1) (Bari and Jones 2009). The availability of synthetic JAs facilitated “spray and pray” approaches to investigate the effects of exogenous compounds in a wide range of plant species. These studies provided evidence that JAs inhibit growth (Aldridge et al. 1971; Staswick et al. 1992), promote senescence (Ueda and Kato 1980), and attract insect pollinators (Baker et al. 1981). Subsequent studies showed that exogenous JAs also induce the accumulation of various stress-related compounds, including proteinase inhibitors (PIs), defensive secondary metabolites, pathogenesis-related proteins, and vegetative storage proteins (Farmer and Ryan 1990; Parthier 1990; Staswick 1990; Gundlach et al. 1992). Newly emerging molecular approaches further demonstrated that low concentrations of exogenous JAs provoke global changes in plant gene expression (Farmer et al. 1992; Sembdner and Parthier 1993; Creelman and Mullet 1997). This early era of jasmonate research established that exogenous JAs elicit growth- and stress-related responses by a mechanism that likely depends on changes in gene expression.

### Quantification of endogenous jasmonates

The ability of any exogenous substance to elicit a biological response is insufficient to establish a role for that compound as a regulatory factor. Rather, it is necessary to demonstrate that specific physiological effects coincide with the spatial and temporal accumulation of the metabolite in plant tissues. The discovery that JAs have signaling properties prompted the development of methods to measure these compounds in plant tissues (Creelman and Mullet 1997). Analytical platforms for quantifying JAs range from high-performance liquid chromatography (Yamane et al. 1981) and qualitative radio immunolabeling (Knöfel et al. 1984) to quantitative GC-MS analyses (Müller et al. 2002) and current state-of-the-art UPLC-MS/MS approaches capable of detecting target metabolites in the pmol range (e.g. Balcke et al. 2012). The low abundance of JAs in plant tissues is consistent with the notion that phytohormones act at concentrations several orders of magnitude below that of most primary metabolites. Analysis using increasingly sensitive instruments revealed the chemical diversity of JAs and related oxylipins (i.e. oxygenated fatty acids) in a broad range of plant species (Creelman and Mullet 1997; Wasternack and Feussner 2018).

### Elucidation of the jasmonate biosynthetic pathway

**Figure 4.**
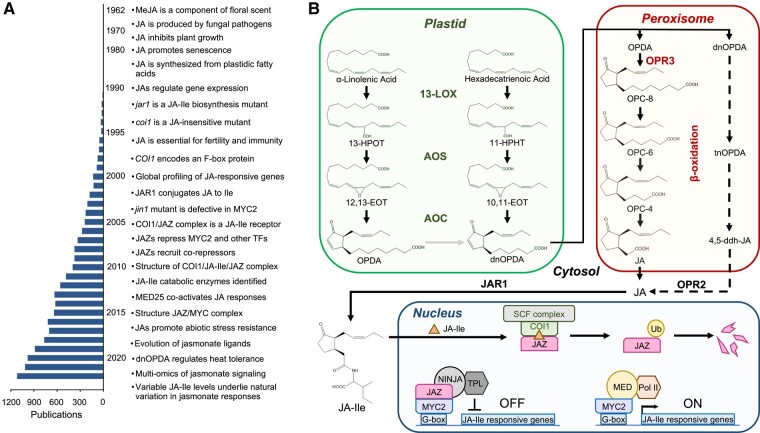
Major advances in elucidating the jasmonate pathway. **A)** Substantial milestones in jasmonate research are listed together with the number of jasmonate-related publications, based on the results of a 2023 PubMed search for “jasmonate.” Due to space constraints, not all milestones are included. See Wasternack (2015) for a comprehensive history of jasmonate research. **B)** Scheme of JA-Ile biosynthesis and signaling. See main text for details. 10,11-EOT, (11*S*)-10,11-epoxy-octadecatrienoic acid; 11-HPHT, 11(*S*)-hydroperoxy-hexadecatrienoic acid; 12,13-EOT, (13*S*)-12,13-epoxy-octadecatrienoic acid; 13-HPOT, 13(*S*)-hydroperoxy-octadecatrienoic acid; 13-LOX, 13-LIPOXYGENASE; 4,5-ddh-JA, 4,5-didehydro JA; AOC, ALLENE OXIDE CYCLASE; AOS, ALLENE OXIDE SYNTHASE; COI1, CORONATINE INSENSITIVE 1; dnOPDA, dinor-oxo-phytodienoic acid; JA, (+)-7-*iso*-jasmonic acid; JA-Ile, (+)-7-*iso*-jasmonoyl-isoleucine; JAZ, JAZMONATE ZIM DOMAIN; MED, MEDIATOR complex; NINJA, NOVEL INTERACTOR OF JAZ; OPC-4, 3-oxo-2-(2-(*Z*)-pentenyl cyclopentane-1-butanoic acid; OPC-6, 3-oxo-2-(2-(*Z*)-pentenyl)-cyclopentane-1-hexanoic acid); OPC-8, 3-oxo-2-(2-(*Z*)-pentenyl)-cyclopentane-1-octanoic acid; OPDA, 12-oxo-phytodienoic acid; OPR2, OPDA REDUCTASE 2; OPR3, OXO-PHYTODIENOIC ACID REDUCTASE 3; Pol II, RNA polymerase II; SCF, SKP1-CUL1-F-box; tnOPDA, tetranor-OPDA; TPL, TOPLESS; Ub, ubiquitin.

A complete understanding of regulatory metabolites requires knowledge of how and where the compound is produced in plant cells. A combination of biochemical and genetic approaches was particularly useful for elucidating the enzymatic reactions that convert polyunsaturated fatty acids to JA and the major receptor-active form of the phytohormone, jasmonoyl-L-isoleucine (JA-Ile) (Browse 2009) (Fig. 4). Classical biochemical fractionation and labeling experiments demonstrated that JA is produced from chloroplastic pools of α-linolenic acid (18:3) via lipoxygenase (LOX) enzymes (Vick and Zimmerman 1984). The elucidation of jasmonate biosynthesis has been extensively reviewed (Wasternack 2015), and the pathway is summarized in Fig. 4. The intracellular location of jasmonate biosynthetic enzymes was validated using immunocytochemical and cell biological approaches (Schaller and Weiler 1997; Wasternack and Feussner 2018). Activity-based purification of several jasmonate biosynthetic enzymes from diverse plant species facilitated the molecular cloning of the corresponding genes using information deduced from the amino acid sequences of the purified proteins (e.g. Song and Brash 1991). These studies confirmed the presence of plastidic or peroxisomal targeting sequences in the relevant enzymes. The development of plant genomic resources, including sequencing of the *Arabidopsis* genome (Arabidopsis Genome Initiative 2000), greatly facilitated efforts to identify genes involved in jasmonate biosynthesis and other aspects of jasmonate biology.

### Mutants galore

The importance of well-defined mutants (e.g. lines harboring null alleles) for elucidating the jasmonate pathway and understanding the functions of JAs cannot be overstated. Identification of jasmonate-deficient *Arabidopsis* mutants in forward genetic screens proved instrumental not only for validating early models of jasmonate biosynthesis but also for uncovering new steps in the pathway (Schaller and Weiler 1997; Browse 2009; Wasternack and Feussner 2018). This approach was facilitated by the easily recognizable phenotypes of JA deficiency in *Arabidopsis*, including male sterility and increased susceptibility to pathogens and insects. The first jasmonate biosynthetic mutant to be identified was *jasmonic acid resistant 1* (*jar1*), which displays insensitivity to JA- and MeJA-inhibited root growth (Staswick et al. 1992). Because these JAs strongly elicit responses in wild-type plants, the most straightforward interpretation at the time was that *jar1* is a jasmonate perception mutant. However, map-based cloning of *JAR1* showed that it encodes a JA-conjugating enzyme responsible for the production of JA-Ile, indicating that *jar1* is in fact a biosynthetic mutant that fails to produce a positive regulator (i.e. JA-Ile) of jasmonate responses (Staswick et al. 2002; Staswick and Tiryaki 2004). JAR1 is a member of the GH3 family of acyl acid amido synthetases, which conjugate amino acids to many phytohormones and likely other regulatory metabolites as well (Staswick and Tiryaki 2004; Westfall et al. 2012). Interestingly, a natural sequence polymorphism abrogating the expression of *JAR1* accounts for much of the variation in induced defense responses within wild populations of *Nicotiana attenuata* (Ray et al. 2023).

Another key advance in understanding the biosynthesis and function of JAs was the discovery that severe JA deficiency in *Arabidopsis* results in male sterility due to defects in pollen development and anther dehiscence (Feys et al. 1994; Browse 2009). The reversibility of this phenotype by exogenous JA was first documented in a fatty acid desaturase triple mutant (*fad3 fad7 fad8*) that fails to produce trienoic fatty acid precursors of JA (McConn and Browse 1996). The application of JA to flower buds of the *fad3 fad7 fad8* mutant recovered its fertility, demonstrating that the conversion of 18:3 to JA is essential for normal flower development. Subsequent screens for male-sterile mutants that could be chemically complemented by JA treatment led to the isolation of additional biosynthetic mutants, including those impaired in ALLENE OXIDE SYNTHASE, 12-OXO-PHYTODIENOIC ACID REDUCTASE 3 (OPR3), and DEFECTIVE IN ANTHER DEHISCENCE1 (DAD1) (reviewed by Browse 2009) (Fig. 4). The latter enzyme is a phospholipase A1 that acts in floral tissues to produce fatty acid precursors of JA from plastidial galactolipids (Ishiguro et al. 2001). In parallel with studies on the role of JAs in male fertility, the availability of JA biosynthetic mutants was instrumental in establishing a causal link between JAs and resistance to a broad spectrum of biotic agents, including arthropod herbivores and necrotrophic pathogens (Glazebrook 2005; Howe and Jander 2008; Bari and Jones 2009; Browse 2009).

Functional analysis of genes encoding biosynthetic enzymes and other components of the jasmonate pathway was advanced through the use of reverse genetic approaches that leveraged newly developed molecular tools, most notably the sequenced *Arabidopsis* genome (Arabidopsis Genome Initiative 2000) and sequence-indexed insertional mutants (Alonso et al. 2003). These resources were particularly important for the characterization of jasmonate biosynthetic enzymes encoded by multigene families, as functional redundancy among paralogs often precludes the identification of mutants in forward genetic screens (Schilmiller et al. 2007; Delfin et al. 2022). The *13-LOX* gene family in *Arabidopsis* exemplifies the use of reverse genetics in helping to decipher the subfunctionalization of specific family members. All 4 13-LOX enzymes (LOX2, LOX3, LOX4, and LOX6) in *Arabidopsis* oxygenate 18:3 and contribute to JA production, likely explaining why *Arabidopsis 13-lox* mutants have not been identified in forward genetic screens. Detailed analysis of single and multiorder *lox* mutants showed that each paralog preferentially acts in different tissues and cell types or is involved in other aspects of oxylipin biosynthesis (Bannenberg et al. 2009; Caldelari et al. 2011; Chauvin et al. 2013; Grebner et al. 2013; Mochizuki et al. 2016). Reverse genetic approaches were also useful for uncovering the existence of alternative pathways that operate in the absence of the major JA biosynthetic route. For example, in mutants lacking OPR3, OPDA can be converted to 4,5-di-dehydro-JA in the peroxisome and then reduced to JA in the cytosol by OPR2 (Fig. 4) (Chini et al. 2018; Howe 2018). Although OPR2 contributes weakly to JA production in *Arabidopsis*, recent evidence suggests that this alternative biosynthetic route predated the OPR3-dependent pathway in the evolution of jasmonate biosynthesis (Chini et al. 2023).

A satisfactory understanding of phytohormones and other regulatory metabolites depends on knowledge obtained from diverse plant species. In general, the roles of JAs in promoting senescence, growth inhibition, and broad-spectrum resistance to herbivores and pathogens are broadly conserved across land plants (Monte et al. 2019). However, whereas JAs appear to be essential for normal reproductive development in all plants examined to date, defects in the JA pathway often give rise to reproductive phenotypes that are specific to a particular species or lineage. The rice *extra glume1* (*eg1*) mutant, which is defective in a DAD1 homolog, is impaired in early stages of spikelet and floret development but show normal stamen and pollen maturation (Cai et al. 2014). Disruption of JA biosynthesis in the maize (*Zea mays*) sex determination mutant *tasselseed 1* (*ts1*) results in feminization of the male inflorescence (tassel) but does not affect early spikelet and floret development (Acosta et al. 2009). The lack of jasmonate responsiveness in the tomato (*Solanum lycopersicum*) *jasmonic acid-insensitive 1* (*jai1*) mutant impairs the maternal control of seed development while decreasing but not eliminating pollen viability (Li et al. 2004; Goetz et al. 2012). These examples illustrate how the study of mutants in diverse plants adds important perspectives to our understanding of jasmonate biology.

### Regulation of jasmonate biosynthesis and catabolism

A substantial advance toward understanding the regulation of jasmonate biosynthesis was the discovery that various stress-related treatments result in the rapid accumulation of endogenous JAs. Examples of eliciting stimuli include the treatment of cultured plant cells with fungal cell wall fragments (Gundlach et al. 1992) and mechanical wounding of leaves (Creelman et al. 1992; Farmer and Ryan 1992). The latter observation linked groundbreaking work on wound-inducible defensive PIs in tomato to the discovery that exogenous JAs rapidly activate the expression of PI-encoding genes (Green and Ryan 1972; Farmer and Ryan 1990; Farmer et al. 1992). A model in which the perception of wounding and other stress-related signals at the cell surface triggers the production of JA and concomitant defense responses has garnered strong support over the past 3 decades (Farmer and Ryan 1992; Howe 2019). These early insights into the roles of JAs in inducing the expression of genes encoding defensive compounds and their accumulation opened up new avenues of investigation into how this lipid-based signaling pathway controls plant immunity (Glazebrook 2005; Karban and Baldwin 2007; Campos et al. 2014; Wang, Wu, et al. 2019).

Owing to its simplicity and effectiveness in inducing jasmonate biosynthesis, mechanical wounding has been used extensively to study the dynamics of jasmonate responses within and between different plant tissues. In general, JA and JA-Ile levels increase within minutes of tissue damage and peak within approximately 1 h (Table 1) (Baldwin et al. 1997; McConn and Browse 1996; Glauser et al. 2009; Koo et al. 2009). Basal levels of OPDA are approximately 500–600 pmol/g fresh weight (FW) in undamaged *Arabidopsis* leaves and 30–40 pmol/g FW in roots and increase 40-fold in leaves and 6-fold in roots after leaf wounding (Table 1). Although basal JA levels are lower than basal OPDA levels and are often below the limit of quantification in undamaged tissues, JA levels can increase up to 500-fold after mechanical wounding. Peak levels of wound-induced JA-Ile are typically much less (e.g. 10-fold) than peak levels of JA in wounded tissues (Table 1). This observation is consistent with the “prohormone” concept in which JA availability is a limiting factor for the JAR1-catalyzed formation of JA-Ile (Staswick and Tiryaki 2004; Acosta and Farmer 2010; Koo 2018). The large differences in the relative abundances of JA biosynthetic precursors and intermediates (18:3 > OPDA > JA > JA-Ile) also illustrate how the tightly regulated biosynthesis of these regulatory metabolites depends on an appropriate supply of precursors along the entire biosynthetic route (Table 1).

**Table 1.** Distribution of jasmonates in *Arabidopsis*

Tissue	Condition	OPDA^a^	JA^a^	JA-Ile^a^	References
Leaves	Resting	600	<10	<2	Schulze et al. (2019), Reymond et al. (2000), Koo et al. (2009)
Leaves	1 h post leaf wounding	25,000	7,000	400	Schulze et al. (2019), Reymond et al. (2000), Koo et al. (2009)
Roots	Resting	35	<10	<2	Schulze et al. 2019
Roots	1 h Post leaf wounding	100	60	60	Schulze et al. 2019

^a^Metabolite levels are in pmol/g FW tissue.

Precise control over the spatial and temporal accumulation of phytohormones depends not only on biosynthetic enzymes but also on factors involved in metabolite transport and catabolism (Li et al. 2021). In the case of JAs, several metabolic transformations are crucial for preventing the overactivation of costly jasmonate-mediated stress responses (Koo and Howe 2009; Heitz et al. 2016; Wasternack and Feussner 2018). For example, cytosolic pools of JA are depleted via the combined action of highly specific JA hydroxylases and sulfotransferases (Caarls et al. 2017; Smirnova et al. 2017; Fernández-Milmanda et al. 2020). JA-Ile itself is inactivated both by hydrolysis of the amide bond (Woldemariam et al. 2012; Widemann et al. 2013; Koo 2018) and by cytochrome P450 enzymes that oxidize the pentenyl side chain to generate the corresponding hydroxy and carboxy derivatives (Koo et al. 2011, 2014; Heitz et al. 2012; Aubert et al. 2015). The finding that JA induces the expression of genes encoding these catabolic enzymes is consistent with the notion of negative feedback regulation of JA-Ile levels (Heitz et al. 2016; Koo 2018). Notably, the rapid inducibility of these and other jasmonate-related genes has been exploited through “guilt-by-associated” coexpression approaches to identify components of the jasmonate response pathway (e.g. Koo et al. 2011).

Homeostasis of plant regulatory metabolites also involves processes that control the systemic production of the chemical signal. The jasmonate pathway provides an excellent model to address this question (Gao et al. 2023; Morin et al. 2023). JA and JA-Ile levels increase rapidly not only locally at the site of injury but also in distal unwounded leaves (Glauser et al. 2009; Koo et al. 2009) and roots (Gasperini et al. 2015). Leaf injury provoked by mechanical wounding or insect herbivory stimulates the long-distance translocation of OPDA and JA to mount defense responses in distal organs (Schulze et al. 2019; Li et al. 2020). Concomitant with this burst of JAs is the rapid propagation of slow wave electrical potentials governed by GLUTAMATE RECEPTOR-LIKE (GLR) proteins (Mousavi et al. 2013; Nguyen et al. 2018). Wounding also stimulates the release of extracellular elicitors such as Glu, which binds to specific GLRs to regulate long-distance Ca^2+^ waves (Toyota et al. 2018; Alfieri et al. 2020; Bellandi et al. 2022). Recent studies in *Arabidopsis* further showed that tissue injury results in the generation of aglucone elicitors of the GLR-dependent propagation of slow wave potentials via glucohydrolase enzymes (Gao et al. 2023). Consistent with these findings, mutants defective in specific GLRs (e.g. GLR3.3 and GLR3.6) are impaired in wound-induced slow wave potentials, long-distance increases in cytosolic Ca^2+^ and jasmonate accumulation in distal leaves (Mousavi et al. 2013; Nguyen et al. 2018; Toyota et al. 2018). Specific DAD1-like lipases appear to link wounding-responsive electrical signaling to the activation of JA biosynthesis (Morin et al. 2023).

### Elucidation of the core jasmonate signaling pathway

By the mid-1990s, it was becoming increasingly evident that the potent effects of JAs on gene expression involve a specific mechanism that links the perception of these compounds to transcriptional reprogramming (Parthier 1990; Farmer and Ryan 1992; Wasternack and Parthier 1997). However, in the absence of any clues into how JAs function, which experimental approaches were best suited to tackle this issue? Once again, mutants identified in genetic screens provided the way forward. The first major advance in understanding the mode of action of JAs came from the identification of the *Arabidopsis coronatine insensitive 1* (*coi1*) mutant. This mutant fails to respond to coronatine, which is a toxin produced by some pathogenic strains of *Pseudomonas syringae* (Feys et al. 1994). As a structural mimic of JA-Ile, exogenous coronatine elicits physiological responses that are very similar to those induced by exogenous JAs. The finding that *coi1* mutants were insensitive to both coronatine and JA indicated that these compounds signal via a similar mechanism (Feys et al. 1994). Importantly, *coi1* mutants are also male sterile and highly susceptible to attack by necrotrophic pathogens and herbivores (Feys et al. 1994; Thomma et al. 1998; Glazebrook 2005). The phenotypic similarity between JA biosynthesis and *coi1* perception mutants provided compelling genetic evidence that endogenous JAs activate a COI1-dependent signaling pathway leading to the expression of jasmonate-responsive genes.

Map-based cloning studies revealed that *COI1* encodes a member of the large (>500 in *Arabidopsis*) F-box family of proteins (Xie et al. 1998). F-box proteins act as specificity determinants in SCF (Skp1/Cullin/F-box)-type E3 ubiquitin ligase complexes that target specific substrates for ubiquitylation and proteolytic destruction (Shabek and Zheng 2014). Accordingly, it was suggested that COI1 recruits a negative regulator of jasmonate responses for targeted degradation, which in turn promotes fertility and defense responses (Xie et al. 1998). This model is supported by the sequence similarity between COI1 and the closely related F-box protein TRANSPORT INHIBITOR RESPONSE 1 (TIR1) and by emerging evidence that TIR1 is a central component of the auxin perception apparatus (Ruegger et al. 1998). An intensive search for COI1 substrates included genetic screens to identify additional regulators of the pathway (Ellis and Turner 2001; Hilpert et al. 2001; Xu et al. 2001; Jensen et al. 2002; Lorenzo et al. 2004), identification of COI1-interacting proteins (Devoto et al. 2002) and cataloging of JA-responsive genes that are expressed in a COI1-dependent manner (Devoto et al. 2005). Despite these efforts, viable candidate COI1 substrates were not identified, and progress toward understanding the molecular mechanism of jasmonate signaling was at an impasse for nearly a decade (Browse 2009). One notable discovery during this period was the identification of jasmonate-insensitive mutants that are defective in a basic helix–loop–helix-type TF called MYC2 (Berger et al. 1996; Boter et al. 2004; Lorenzo et al. 2004). Although MYC2 and its closely related proteins (e.g. MYC3 and MYC4 in *Arabidopsis*) are regarded as master positive regulators of jasmonate responses (Fernández-Calvo et al. 2011; Kazan and Manners 2013), a satisfactory model to explain how MYC activity is linked to jasmonate and COI1 failed to emerge at this time.

In 2007, the discovery of JASMONATE ZIM DOMAIN (JAZ) proteins as the elusive COI1 substrates was achieved through the analysis of gene coexpression modules (Thines et al. 2007; Yan et al. 2007) and positional cloning of a mutated gene that confers dominant insensitivity to JA (Chini et al. 2007). Thines and coworkers found that 8 related genes encoding uncharacterized proteins containing a ZINC-FINGER PROTEIN EXPRESSED IN INFLORESCENCE MERISTEM (ZIM) motif were rapidly induced by exogenous jasmonate (Thines et al. 2007). Similarly, genome-wide expression profiling showed that several wound-induced transcripts encoding these ZIM domain-containing proteins accumulated in a JA-dependent manner (Yan et al. 2007). Finally, the causative mutation underlying the JA-insensitive *jai3-1* mutant phenotype was shown to reside in a chromosomal region that contains a member (*JAZ3*) of the same gene family (Chini et al. 2007). This plant-specific gene family was named *JAZ*, with 13 recognized members in *Arabidopsis* (Chini et al. 2007; Thines et al. 2007; Yan et al. 2007; Thireault et al. 2015). The size of the rapidly evolving *JAZ* family ranges from one in the common liverwort (*Marchantia polymorpha*) to more than 20 members in many flowering plants (Howe et al. 2018; Monte et al. 2019).

Three key findings associated with the discovery of JAZ helped to fill in the remaining major gaps in understanding the core mechanism of jasmonate signaling. First, mutated JAZ proteins that fail to interact with COI1 strongly repress the expression of jasmonate-responsive genes (Chini et al. 2007; Thines et al. 2007; Yan et al. 2007). Second, JAZ proteins exert repression by binding directly to MYC TFs (Chini et al. 2007) and many other TFs as well (Fernández-Calvo et al. 2011; Chini et al. 2016; Howe et al. 2018). Finally, the physical interaction between COI1 and JAZ is promoted by JA-Ile but not its metabolic precursors or derivatives, including OPDA, JA, and MeJA; it was therefore concluded that a COI1–JAZ complex constitutes the site of JA-Ile perception (Thines et al. 2007). These collective findings provided a basis for a model (Fig. 4) in which MYC-dependent responses are repressed by JAZ in the absence of JA-Ile. In response to stress-related or developmental cues that stimulate JA-Ile accumulation, JA-Ile promotes the SCF^COI1^-dependent turnover of JAZ and subsequent expression of jasmonate-responsive genes.

Follow-up studies showed that coronatine also promotes the formation of COI1–JAZ complexes and that the C-terminal Jas motif of JAZ harbors a “degron” sequence that mediates the hormone-dependent COI1–JAZ interaction (Katsir et al. 2008; Melotto et al. 2008). Mutation of the degron motif stabilizes JAZ against hormone-dependent degradation, resulting in dominant repression of jasmonate responses (Chini et al. 2007; Thines et al. 2007; Yan et al. 2007; Melotto et al. 2008). Biochemical and structural analyses further showed that the COI1–JAZ interaction is mediated specifically by (+)-7-*iso*-JA-Ile, which is the major JA-Ile stereoisomer in plant tissues (Fonseca et al. 2009; Sheard et al. 2010). In validating and extending the pioneering work on JAR1 (Staswick and Tiryaki 2004), it is now evident that jasmonate responses elicited by exogenous JA and MeJA require metabolic transformation of these compounds to (+)-7-*iso*-JA-Ile in plant tissues.

### Structural insights into jasmonate perception and signaling

The discovery that COI1–JAZ complexes are a high-affinity receptor for JA-Ile (Thines et al. 2007) was aided by the molecular glue paradigm, which first emerged from the study of auxin perception (Dharmasiri et al. 2003; Kepinski and Leyser 2005; Tan et al. 2007). The 3-dimensional structure of the ligand-bound COI1–JAZ ternary complex shows that an inositol pyrophosphate (likely InsP_8_) serves as a COI1 structural cofactor and that the JAZ degron simultaneously interacts with both the bound hormone and COI1 (Fonseca et al. 2009; Sheard et al. 2010; Laha et al. 2015). This model of COI1–JAZ as a high-affinity coreceptor does not exclude the possibility that JA-Ile first binds to COI1 with low affinity, followed by the formation of a high-affinity COI1–JAZ complex. This 2-step model of JA-Ile perception has received experimental support by showing that coronatine weakly interacts (i.e. low micromolar affinity) with COI1 in the absence of JAZ (Yan et al. 2018). Similar results were recently reported for TIR1, which can bind auxin at low micromolar affinity in the absence of an Aux/IAA substrate (Cao et al. 2022). These collective findings support a scenario in which molecular glue-type hormones were recruited as signals based on their ability to promote protein–protein interactions (Fig. 3).

The structural elucidation of MYC3 binding to the Jas domain of JAZ9 further clarified the molecular mechanism by which JAZ proteins repress MYC TFs (Zhang et al. 2015). In the absence of the hormone, the Jas motif assumes an α-helical structure that intercalates within the MYC N-terminal fold, thereby inhibiting the interaction of MYC with the MED25 subunit of the mediator complex (Çevik et al. 2012; Chen et al. 2012; Zhang et al. 2015). JAZ proteins recruit transcriptional corepressors of the TOPLESS family via the adaptor protein NINJA (Pauwels et al. 2010) and, in some cases, directly interact with TOPLESS through an EAR motif (Kagale et al. 2010; Shyu et al. 2012). JA-Ile-induced JAZ degradation releases MYCs from repression while simultaneously facilitating the recruitment of coactivators such as MED25 (Zhang et al. 2015; An et al. 2017) (Fig. 4). Thus, JAZ proteins perform dual functions as transcriptional repressors in the absence of JA-Ile and as coreceptors in the presence of the hormone. Understanding the structural and dynamic behavior of JAZ-associated transcriptional complexes represents an important future challenge (Zhang et al. 2017; Zhou et al. 2023). The prolific ability of JAZs to interact with proteins in other signaling pathways (Robert-Seilaniantz et al. 2011; Song et al. 2014; Berens et al. 2017; Howe et al. 2018; Zhou et al. 2023) provides a window into how regulatory metabolites connect pathways to reconfigure transcriptional programs in a flexible and dynamic manner.

### Multiple layers of repression

A recurring theme in hormone biology is the existence of mechanisms to attenuate the amplitude and duration of responses through negative feedback control (Fig. 1). In the case of jasmonate, overactivation of the pathway results in stunted growth, decreased fecundity, loss of photosynthetic capacity, and, in its extreme, cell death (Baldwin 1998; Yan et al. 2007; Zhang and Turner 2008; Guo et al. 2018, 2022). Among the major mechanisms to attenuate these costly responses are the catabolism of bioactive JAs (see above) and de novo synthesis of JAZ repressors. The latter mechanism is based on the observation that *JAZ* genes are rapidly and strongly expressed by MYC2 in response to jasmonate (Chini et al. 2007; Thines et al. 2007; Chung et al. 2008; Moreno et al. 2013; Zander et al. 2020). Effective JAZ-mediated repression relies on the recruitment of various corepressor and adaptor proteins, including NINJA, TOPLESS, and epigenetic modifying enzymes (Pauwels et al. 2010; Howe et al. 2018; Zander 2021). JAZ stabilization resulting from alternative splicing events that eliminate or weaken COI1 interactions, as well as jasmonate-induced expression of factors that compete with MYCs for DNA binding, represent additional layers of negative feedback control (Chung et al. 2010; Nakata et al. 2013; Fonseca et al. 2014; Qi et al. 2015; Zhang et al. 2017; Wu et al. 2020). These many layers of signal attenuation highlight the negative fitness consequences of overactivating jasmonate responses.

### Evolution of the jasmonate pathway

Functional phylogenomic approaches based on recently sequenced streptophyte (charophyte algae and land plants) genomes have provided important new insights into processes that shaped the emergence of metabolic and signaling pathways during the colonization of land by plants (Maeda and Fernie 2021; Bowman 2022; Rieseberg et al. 2023). Recent studies on the evolution of JAs have contributed to these efforts by revealing lineage-specific innovations in both JA biosynthetic and signaling pathways. Genome analysis revealed that bryophyte lineages (hornworts, liverworts, and mosses) contain orthologs for all core signaling JA components, including COI1, JAZ, NINJA, TPL, and MYC (Bowman et al. 2017; Monte et al. 2018). Functional analyses of the COI1-JAZ-MYC signaling module showed that the jasmonate pathway likely emerged in the common ancestor of land plants more than 450 million years ago (Bowman et al. 2017; Peñuelas et al. 2019; Chini et al. 2023). Interestingly, however, bryophytes neither synthesize nor respond to JA-Ile, which is the receptor-active form of the hormone in vascular plants. Rather, studies with *M. polymorpha* (Mp) and the lycophyte *Huperzia selago* identified several isomers of dnOPDA (dn-cis-OPDA and dn-iso-OPDA) as active ligands in these species (Monte et al. 2018; Kneeshaw et al. 2022). The selectivity of the MpCOI1–JAZ coreceptor for dnOPDA ligands was attributed to a single amino acid change in MpCOI1 that alters the size of the ligand-binding pocket, as well as specific residues in the Jas motif of MpJAZ. A broad survey of the green lineage recently confirmed and extended these insights into the evolutionary trajectory of JAs (Chini et al. 2023).

Functional phylogenomic studies have also provided insight into the extent to which the physiological functions of JAs are conserved in land plants. Transcriptional changes in *M. polymorpha* induced by treatment with dnOPDA are similar to transcriptional changes elicited by JA-Ile in flowering plants, including the induction of genes related to secondary metabolism and wounding responses (Devoto et al. 2005; Chini et al. 2018). However, whereas JA-Ile promotes defense and inhibits growth and fertility in *Arabidopsis*, dnOPDA appears to regulate defense and growth responses but not fertility in *M. polymorpha* (Monte et al. 2018). Given the reduced fertility of OPDA-deficient mutants of the moss *Physcomitrium patens* (Stumpe et al. 2010), additional work is needed to better understand the evolutionary basis of the roles of JAs in plant reproductive processes.

It is noteworthy that cyclopentenone JAs such as OPDA and dnOPDA are reactive electrophilic compounds that exhibit signaling properties independently of COI1. A recent study provided evidence that these compounds signal through a COI1-independent pathway to promote thermotolerance in diverse species, including *Arabidopsis*, *M. polymorpha*, and the charophyte alga *Klebsormidium nitens* (Monte et al. 2020). These findings suggest an ancestral role for JAs in regulating protection against heat stress, which was likely important for plant colonization of terrestrial habitats. Elucidation of the COI1-independent signaling pathway by which cyclopentanone JAs promote thermotolerance promises to provide new insight into how the larger family of lipid-derived JAs were recruited into distinct signaling pathways during plant evolution.

## Concluding remarks and future perspectives

The extraordinary diversity of plant metabolism arises from adaptive processes such as the neofunctionalization of duplicated enzyme-encoding genes and increased enzyme promiscuity (Chae et al. 2014; Weng et al. 2021). As a consequence, hundreds of thousands of secondary metabolites constitute an evolving chemical “library” from which to recruit small molecules into roles that promote plant fitness in dynamic and often harsh environments. This process is exemplified by small-molecule plant hormones, which have acquired the ability to modulate cellular processes needed to help the plant adapt its growth and development to changing conditions. Currently recognized phytohormones appear to be part of a larger and more diverse group of chemical mediators whose action is linked in part to transcriptional control. Lessons from the study of the jasmonate pathway and other phytohormones provide a guide to drive future progress in unveiling the physiological roles of regulatory metabolites, their modes of perception and signaling, and their evolutionary history. Current knowledge gaps (see Outstanding Questions) pose exciting challenges for the future of plant science, which may help to inform efforts to modify signaling pathways for crop improvement.

A major bottleneck in understanding how plant metabolites influence gene expression and associated traits is the lack of insight into the direct targets of these compounds. The vast majority of small-molecule receptors in plants were identified through the use of mutants that are unresponsive to a defined eliciting cue, including (pro)hormones and hormone agonists. Following the identification of such mutants, molecular genetic techniques are employed to track down the causal mutation. The strength of this approach lies in linking a specific gene to a robust biological response, as well as its application to any bioactive compound whose chemical identity is known. Moreover, advances in genome-sequencing technology have greatly accelerated the process of identifying the mutated gene underlying a phenotype of interest (e.g. Schneeberger et al. 2009). Limitations of the classical genetic approach include the labor intensiveness of genetic screens and the possibility that perception mutants are difficult to identify due to functional redundancy within receptor-encoding gene families.

These considerations justify future research to systemically investigate regulatory metabolite–protein interactions in plants. Such efforts have historically been hampered by technical challenges related to the transient, low-affinity nature of these bimolecular associations. Nevertheless, recent advances in mass spectrometry have facilitated the development of powerful approaches to identify biologically relevant protein–metabolite interactions in various model organisms (Piazza et al. 2018; Luzarowski and Skirycz 2019). This technology can be applied in either a non-targeted or targeted manner (i.e. identification of proteins that bind to a specific metabolite) and is adaptable for use in plants (Schlossarek et al. 2023). Improvements in molecular modeling and other computational approaches for predicting protein–metabolite interactions, which are widely used for drug discovery (Lavecchia 2015), are also expected to be employed to identify candidate protein targets of plant regulatory metabolites. In the coming years, there is much to look forward to as these new technologies are combined with robust genetic approaches to delve deeper into the expanding universe of plant regulatory metabolites.

**OUTSTANDING QUESTIONS**

- What are the direct targets of the myriad volatile and nonvolatile plant secondary metabolites that exhibit signaling attributes?
- To what extent can untargeted mass spectrometry-based and predictive approaches be used to systematically identify protein–metabolite interactions with a wide range of affinities?
- How do evolutionary forces recruit secondary metabolites into signaling pathways?
- Beyond the small number of plant hormones known to act in the nucleus, to what extent do small molecules interact with and modulate the activities of transcriptional regulators?
- How can knowledge of regulatory metabolites be used to develop crop varieties that are more productive, nutritious, and better adapted to changing climate conditions?

## Dive Curated Terms

The following phenotypic, genotypic, and functional terms are of significance to the work described in this paper:

LOX2 Gramene: AT3G45140
LOX2 Araport: AT3G45140

OPR2 Gramene: AT1G76690
OPR2 Araport: AT1G76690

OPR3 Gramene: AT2G06050
OPR3 Araport: AT2G06050

ALLENE CHEBI: CHEBI:37601

hormone CHEBI: CHEBI:24621

MED25 Gramene: Solyc12g070100
MED25 Araport: Solyc12g070100

plant hormone CHEBI: CHEBI:37848

MYC3 Gramene: AT5G46760
MYC3 Araport: AT5G46760

aos Gramene: AT5G42650
aos Araport: AT5G42650

LOX3 Gramene: At1g17420
LOX3 Araport: At1g17420

JAR1 Gramene: AT2G46370
JAR1 Araport: AT2G46370

JAZ3 Gramene: AT3G17860
JAZ3 Araport: AT3G17860

COI1 Gramene: AT2G39940
COI1 Araport: AT2G39940

TPL Gramene: At1g15750
TPL Araport: At1g15750

trans-zeatin CHEBI: CHEBI:16522

NINJA Gramene: AT4G28910
NINJA Araport: AT4G28910

## References

[kiae045-B1] Acosta IF , Farmer EE. Jasmonates. Arabidopsis Book. 2010:8:e0129. 10.1199/tab.0129 22303255 PMC3244945

[kiae045-B2] Acosta IF , Laparra H, Romero SP, Schmelz E, Hamberg M, Mottinger JP, Moreno MA, Dellaporta SL. *Tasselseed1* is a lipoxygenase affecting jasmonic acid signaling in sex determination of maize. Science. 2009:323(5911):262–265. 10.1126/science.1164645 19131630

[kiae045-B3] Aldridge DC , Galt S, Giles D, Turner WB. Metabolites of *Lasiodiplodia theobromae*. J Chem Soc. 1971:1971:1623–1627. 10.1039/j39710001623

[kiae045-B4] Alfieri A , Doccula FG, Pederzoli R, Grenzi M, Bonza MC, Luoni L, Candeo A, Romano Armada N, Barbiroli A, Valentini G, et al The structural bases for agonist diversity in an *Arabidopsis thaliana* glutamate receptor-like channel. Proc Natl Acad Sci U S A. 2020:117(1):752–760. 10.1073/pnas.1905142117 31871183 PMC6955363

[kiae045-B5] Alonso JM , Stepanova AN, Leisse TJ, Kim CJ, Chen H, Shinn P, Stevenson DK, Zimmerman J, Barajas P, Cheuk R, et al Genome-wide insertional mutagenesis of *Arabidopsis thaliana*. Science. 2003:301(5633):653–657. 10.1126/science.1086391 12893945

[kiae045-B6] Altmann M , Altmann S, Rodriguez PA, Weller B, Elorduy Vergara L, Palme J, Marín-de la Rosa N, Sauer M, Wenig M, Villaécija-Aguilar JA, et al Extensive signal integration by the phytohormone protein network. Nature. 2020:583(7815):271–276. 10.1038/s41586-020-2460-0 32612234

[kiae045-B7] An C , Li L, Zhai Q, You Y, Deng L, Wu F, Chen R, Jiang H, Wang H, Chen Q, et al Mediator subunit MED25 links the jasmonate receptor to transcriptionally active chromatin. Proc Natl Acad Sci U S A. 2017:114(42):E8930–E8939. 10.1073/pnas.1710885114 28973940 PMC5651773

[kiae045-B8] Anastasiou E , Kenz S, Gerstung M, MacLean D, Timmer J, Fleck C, Lenhard M. Control of plant organ size by KLUH/CYP78A5-dependent intercellular signaling. Dev Cell. 2007:13(6):843–856. 10.1016/j.devcel.2007.10.001 18061566

[kiae045-B9] Arabidopsis Genome Initiative . Analysis of the genome sequence of the flowering plant *Arabidopsis thaliana*. Nature. 2000:408(6814):796–815. 10.1038/35048692 11130711

[kiae045-B10] Aratani Y , Uemura T, Hagihara T, Matsui K, Toyota M. Green leaf volatile sensory calcium transduction in *Arabidopsis*. Nat Commun. 2023:14(1):6236. 10.1038/s41467-023-41589-9 37848440 PMC10582025

[kiae045-B11] Aubert Y , Widemann E, Miesch L, Pinot F, Heitz T. CYP94-mediated jasmonoyl-isoleucine hormone oxidation shapes jasmonate profiles and attenuates defence responses to *Botrytis cinerea* infection. J Exp Bot. 2015:66(13):3879–3892. 10.1093/jxb/erv190 25903915 PMC4473988

[kiae045-B12] Bai Y , Fernández-Calvo P, Ritter A, Huang AC, Morales-Herrera S, Bicalho KU, Karady M, Pauwels L, Buyst D, Njo M, et al Modulation of *Arabidopsis* root growth by specialized triterpenes. New Phytol. 2021:230(1):228–243. 10.1111/nph.17144 33616937

[kiae045-B13] Bailey-Serres J , Parker JE, Ainsworth EA, Oldroyd GED, Schroeder JI. Genetic strategies for improving crop yields. Nature. 2019:575(7781):109–118. 10.1038/s41586-019-1679-0 31695205 PMC7024682

[kiae045-B14] Baker TC , Nishida R, Roelofs WL. Close-range attraction of female oriental fruit moths to herbal scent of male hairpencils. Science. 1981:214(4527):1359–1361. 10.1126/science.214.4527.1359 17812262

[kiae045-B15] Balcke GU , Handrick V, Bergau N, Fichtner M, Henning A, Stellmach H, Tissier A, Hause B, Frolov A. An UPLC-MS/MS method for highly sensitive high-throughput analysis of phytohormones in plant tissues. Plant Methods. 2012:8(1):47. 10.1186/1746-4811-8-47 23173950 PMC3573895

[kiae045-B16] Baldwin IT . Jasmonate-induced responses are costly but benefit plants under attack in native populations. Proc Natl Acad Sci U S A. 1998:95(14):8113–8118. 10.1073/pnas.95.14.8113 9653149 PMC20938

[kiae045-B17] Baldwin IT , Zhang ZP, Diab N, Ohnmeiss TE. Quantification, correlations and manipulations of wound-induced changes in jasmonic acid and nicotine in *Nicotiana sylvestris*. Planta. 1997:201(4):397–404. 10.1007/s004250050082

[kiae045-B18] Bannenberg G , Martínez M, Hamberg M, Castresana C. Diversity of the enzymatic activity in the lipoxygenase gene family of *Arabidopsis thaliana*. Lipids. 2009:44(2):85–95. 10.1007/s11745-008-3245-7 18949503

[kiae045-B19] Bari R , Jones JDG. Role of plant hormones in plant defence responses. Plant Mol Biol. 2009:69(4):473–488. 10.1007/s11103-008-9435-0 19083153

[kiae045-B20] Bellandi A , Papp D, Breakspear A, Joyce J, Johnston MG, de Keijzer J, Raven EC, Ohtsu M, Vincent TR, Miller AJ, et al Diffusion and bulk flow of amino acids mediate calcium waves in plants. Sci Adv. 2022:8(42):eabo6693. 10.1126/sciadv.abo6693 36269836 PMC9586480

[kiae045-B21] Bennett T , Leyser O. The auxin question: a philosophical overview. In: Zažímalová E, Petrášek J, Benková E, editors. Auxin and its role in plant development. Vienna: Springer; 2014. p. 3–19.

[kiae045-B22] Berens ML , Berry HM, Mine A, Argueso CT, Tsuda K. Evolution of hormone signaling networks in plant defense. Annu Rev Phytopathol. 2017:55(1):401–425. 10.1146/annurev-phyto-080516-035544 28645231

[kiae045-B23] Berger S , Bell E, Mullet JE. Two methyl jasmonate-insensitive mutants show altered expression of AtVsp in response to methyl jasmonate and wounding. Plant Physiol. 1996:111:525–531. 12226307 10.1104/pp.111.2.525 PMC157863

[kiae045-B24] Blázquez MA , Nelson DC, Weijers D. Evolution of plant hormone response pathways. Annu Rev Plant Biol. 2020:71(1):327–353. 10.1146/annurev-arplant-050718-100309 32017604

[kiae045-B25] Boter M , Ruíz-Rivero O, Abdeen A, Prat S. Conserved MYC transcription factors play a key role in jasmonate signaling both in tomato and *Arabidopsis*. Genes Dev. 2004:18(13):1577–1591. 10.1101/gad.297704 15231736 PMC443520

[kiae045-B26] Bowman JL . The origin of a land flora. Nat Plants. 2022:8(12):1352–1369. 10.1038/s41477-022-01283-y 36550365

[kiae045-B27] Bowman JL , Kohchi T, Yamato KT, Jenkins J, Shu S, Ishizaki K, Yamaoka S, Nishihama R, Nakamura Y, Berger F, et al Insights into land plant evolution garnered from the *Marchantia* polymorpha genome. Cell. 2017:171(2):287–304.e15. 10.1016/j.cell.2017.09.030 28985561

[kiae045-B28] Brown DE , Rashotte AM, Murphy AS, Normanly J, Tague BW, Peer WA, Taiz L, Muday GK. Flavonoids act as negative regulators of auxin transport in vivo in arabidopsis. Plant Physiol. 2001:126(2):524–535. 10.1104/pp.126.2.524 11402184 PMC111146

[kiae045-B29] Browse J . The power of mutants for investigating jasmonate biosynthesis and signaling. Phytochemistry. 2009:70(13–14):1539–1546. 10.1016/j.phytochem.2009.08.004 19740496

[kiae045-B30] Bunsick M , McCullough R, McCourt P, Lumba S. Plant hormone signaling: is upside down right side up? Curr Opin Plant Biol. 2021:63:102070. 10.1016/j.pbi.2021.102070 34166978

[kiae045-B31] Caarls L , Elberse J, Awwanah M, Ludwig NR, de Vries M, Zeilmaker T, Van Wees SCM, Schuurink RC, Van den Ackerveken G. *Arabidopsis* JASMONATE-INDUCED OXYGENASES down-regulate plant immunity by hydroxylation and inactivation of the hormone jasmonic acid. Proc Natl Acad Sci U S A. 2017:114(24):6388–6393. 10.1073/pnas.1701101114 28559313 PMC5474790

[kiae045-B32] Cai J , Aharoni A. Amino acids and their derivatives mediating defense priming and growth tradeoff. Curr Opin Plant Biol. 2022:69:102288. 10.1016/j.pbi.2022.102288 35987012

[kiae045-B33] Cai Q , Yuan Z, Chen M, Yin C, Luo Z, Zhao X, Liang W, Hu J, Zhang D. Jasmonic acid regulates spikelet development in rice. Nat Commun. 2014:5(1):3476. 10.1038/ncomms4476 24647160

[kiae045-B34] Caldelari D , Wang G, Farmer EE, Dong X. *Arabidopsis lox3 lox4* double mutants are male sterile and defective in global proliferative arrest. Plant Mol Biol. 2011:75(1–2):25–33. 10.1007/s11103-010-9701-9 21052784

[kiae045-B35] Campos ML , Kang J-H, Howe GA. Jasmonate-triggered plant immunity. J Chem Ecol. 2014:40(7):657–675. 10.1007/s10886-014-0468-3 24973116 PMC4143990

[kiae045-B36] Cao S , Kang S, Mao H, Yao J, Gu L, Zheng N. Defining molecular glues with a dual-nanobody cannabidiol sensor. Nat Commun. 2022:13(1):815. 10.1038/s41467-022-28507-1 35145136 PMC8831599

[kiae045-B37] Çevik V , Kidd BN, Zhang P, Hill C, Kiddle S, Denby KJ, Holub EB, Cahill DM, Manners JM, Schenk PM, et al MEDIATOR25 acts as an integrative hub for the regulation of jasmonate-responsive gene expression in *Arabidopsis*. Plant Physiol. 2012:160(1):541–555. 10.1104/pp.112.202697 22822211 PMC3440227

[kiae045-B38] Chae L , Kim T, Nilo-Poyanco R, Rhee SY. Genomic signatures of specialized metabolism in plants. Science. 2014:344(6183):510–513. 10.1126/science.1252076 24786077

[kiae045-B39] Chauvin A , Caldelari D, Wolfender J-L, Farmer EE. Four 13-lipoxygenases contribute to rapid jasmonate synthesis in wounded *Arabidopsis thaliana* leaves: a role for lipoxygenase 6 in responses to long-distance wound signals. New Phytol. 2013:197(2):566–575. 10.1111/nph.12029 23171345

[kiae045-B40] Chen R , Jiang H, Li L, Zhai Q, Qi L, Zhou W, Liu X, Li H, Zheng W, Sun J, et al The *Arabidopsis* mediator subunit MED25 differentially regulates jasmonate and abscisic acid signaling through interacting with the MYC2 and ABI5 transcription factors. Plant Cell. 2012:24(7):2898–2916. 10.1105/tpc.112.098277 22822206 PMC3426122

[kiae045-B41] Chini A , Fonseca S, Fernández G, Adie B, Chico JM, Lorenzo O, García-Casado G, López-Vidriero I, Lozano FM, Ponce MR, et al The JAZ family of repressors is the missing link in jasmonate signalling. Nature. 2007:448(7154):666–671. 10.1038/nature06006 17637675

[kiae045-B42] Chini A , Gimenez-Ibanez S, Goossens A, Solano R. Redundancy and specificity in jasmonate signalling. Curr Opin Plant Biol. 2016:33:147–156. 10.1016/j.pbi.2016.07.005 27490895

[kiae045-B43] Chini A , Monte I, Zamarreño AM, García-Mina JM, Solano R. Evolution of the jasmonate ligands and their biosynthetic pathways. New Phytol. 2023:238(5):2236–2246. 10.1111/nph.18891 36942932

[kiae045-B44] Chini A , Monte I, Zamarreño AM, Hamberg M, Lassueur S, Reymond P, Weiss S, Stintzi A, Schaller A, Porzel A, et al An OPR3-independent pathway uses 4,5-didehydrojasmonate for jasmonate synthesis. Nat Chem Biol. 2018:14(2):171–178. 10.1038/nchembio.2540 29291349

[kiae045-B45] Chung HS , Cooke TF, Depew CL, Patel LC, Ogawa N, Kobayashi Y, Howe GA. Alternative splicing expands the repertoire of dominant JAZ repressors of jasmonate signaling. Plant J. 2010:63(4):613–622. 10.1111/j.1365-313X.2010.04265.x 20525008 PMC2966510

[kiae045-B46] Chung HS , Koo AJK, Gao X, Jayany S, Thines B, Jones AD, Howe GA. Regulation and function of *Arabidopsis JASMONATE-ZIM*-domain genes in response to wounding and herbivory. Plant Physiol. 2008:146(3):952–964. 10.1104/pp.107.115691 18223147 PMC2259048

[kiae045-B47] Creelman RA , Mullet JE. Biosynthesis and action of jasmonates in plants. Annu Rev Plant Physiol Plant Mol Biol. 1997:48(1):355–381. 10.1146/annurev.arplant.48.1.355 15012267

[kiae045-B48] Creelman RA , Tierney ML, Mullet JE. Jasmonic acid/methyl jasmonate accumulate in wounded soybean hypocotyls and modulate wound gene expression. Proc Natl Acad Sci U S A. 1992:89(11):4938–4941. 10.1073/pnas.89.11.4938 1594598 PMC49203

[kiae045-B49] Dani KGS , Loreto F. Plant volatiles as regulators of hormone homeostasis. New Phytol. 2022:234(3):804–812. 10.1111/nph.18035 35170033

[kiae045-B50] Daryanavard H , Postiglione AE, Mühlemann JK, Muday GK. Flavonols modulate plant development, signaling, and stress responses. Curr Opin Plant Biol. 2023:72:102350. 10.1016/j.pbi.2023.102350 36870100 PMC10372886

[kiae045-B51] Davies PJ . The plant hormones: their nature, occurrence, and functions. In: Davies PJ, editor. Plant hormones: biosynthesis, signal transduction, action!. Dordrecht, Netherlands: Springer; 2010. p. 1–15.

[kiae045-B52] Delfin JC , Kanno Y, Seo M, Kitaoka N, Matsuura H, Tohge T, Shimizu T. AtGH3.10 is another jasmonic acid-amido synthetase in *Arabidopsis thaliana*. Plant J. 2022:110(4):1082–1096. 10.1111/tpj.15724 35247019

[kiae045-B53] Demole E , Lederer E, Mercier D. Isolement et détermination de la structure du jasmonate de méthyle, constituant odorant caractéristique de l’essence de jasmin. Helv Chim Acta. 1962:45(2):675–685. 10.1002/hlca.19620450233

[kiae045-B54] Devoto A , Ellis C, Magusin A, Chang H-S, Chilcott C, Zhu T, Turner JG. Expression profiling reveals COI1 to be a key regulator of genes involved in wound- and methyl jasmonate-induced secondary metabolism, defence, and hormone interactions. Plant Mol Biol. 2005:58(4):497–513. 10.1007/s11103-005-7306-5 16021335

[kiae045-B55] Devoto A , Nieto-Rostro M, Xie D, Ellis C, Harmston R, Patrick E, Davis J, Sherratt L, Coleman M, Turner JG. COI1 links jasmonate signalling and fertility to the SCF ubiquitin-ligase complex in *Arabidopsis*. Plant J. 2002:32(4):457–466. 10.1046/j.1365-313X.2002.01432.x 12445118

[kiae045-B56] Dharmasiri N , Dharmasiri S, Jones AM, Estelle M. Auxin action in a cell-free system. Curr Biol. 2003:13(16):1418–1422. 10.1016/S0960-9822(03)00536-0 12932326

[kiae045-B57] Dinneny J , Al-Babili S, Benfey PN. β-Cyclocitral is a conserved root growth regulator. Proc Natl Acad Sci U S A. 2019:116(21):10563–10567. 10.1073/pnas.1821445116 31068462 PMC6534974

[kiae045-B58] Durán-Medina Y , Ruiz-Cortés BE, Guerrero-Largo H, Marsch-Martínez N. Specialized metabolism and development: an unexpected friendship. Curr Opin Plant Biol. 2021:64:102142. 10.1016/j.pbi.2021.102142 34856480

[kiae045-B59] Ellis C , Turner JG. The *Arabidopsis* mutant *cev1* has constitutively active jasmonate and ethylene signal pathways and enhanced resistance to pathogens. Plant Cell. 2001:13(5):1025–1033. 10.1105/tpc.13.5.1025 11340179 PMC135553

[kiae045-B60] Engelberth J , Alborn HT, Schmelz EA, Tumlinson JH. Airborne signals prime plants against insect herbivore attack. Proc Natl Acad Sci U S A. 2004:101(6):1781–1785. 10.1073/pnas.0308037100 14749516 PMC341853

[kiae045-B61] Erb M , Kliebenstein DJ. Plant secondary metabolites as defenses, regulators, and primary metabolites: the blurred functional trichotomy. Plant Physiol. 2020:184(1):39–52. 10.1104/pp.20.00433 32636341 PMC7479915

[kiae045-B62] Erb M , Veyrat N, Robert CAM, Xu H, Frey M, Ton J, Turlings TCJ. Indole is an essential herbivore-induced volatile priming signal in maize. Nat Commun. 2015:6(1):6273. 10.1038/ncomms7273 25683900 PMC4339915

[kiae045-B63] Fàbregas N , Fernie AR. The reliance of phytohormone biosynthesis on primary metabolite precursors. J Plant Physiol. 2021:268:153589. 10.1016/j.jplph.2021.153589 34896926

[kiae045-B64] Farmer EE , Johnson RR, Ryan CA. Regulation of expression of proteinase inhibitor genes by methyl jasmonate and jasmonic acid. Plant Physiol. 1992:98(3):995–1002. 10.1104/pp.98.3.995 16668777 PMC1080300

[kiae045-B65] Farmer EE , Ryan CA. Interplant communication: airborne methyl jasmonate induces synthesis of proteinase inhibitors in plant leaves. Proc Natl Acad Sci U S A. 1990:87(19):7713–7716. 10.1073/pnas.87.19.7713 11607107 PMC54818

[kiae045-B66] Farmer EE , Ryan CA. Octadecanoid precursors of jasmonic acid activate the synthesis of wound-inducible proteinase inhibitors. Plant Cell. 1992:4(2):129–134. 10.2307/3869566 12297644 PMC160114

[kiae045-B67] Felemban A , Moreno JC, Mi J, Ali S, Sham A, AbuQamar SF, Al-Babili S. The apocarotenoid β-ionone regulates the transcriptome of *Arabidopsis thaliana* and increases its resistance against *Botrytis cinerea*. Plant J. 2023:117(2):541–560. 37932864 10.1111/tpj.16510

[kiae045-B68] Fernández-Calvo P , Chini A, Fernández-Barbero G, Chico J-M, Gimenez-Ibanez S, Geerinck J, Eeckhout D, Schweizer F, Godoy M, Franco-Zorrilla JM, et al The *Arabidopsis* bHLH transcription factors MYC3 and MYC4 are targets of JAZ repressors and act additively with MYC2 in the activation of jasmonate responses. Plant Cell. 2011:23(2):701–715. 10.1105/tpc.110.080788 21335373 PMC3077776

[kiae045-B69] Fernández-Milmanda GL , Crocco CD, Reichelt M, Mazza CA, Köllner TG, Zhang T, Cargnel MD, Lichy MZ, Fiorucci A-S, Fankhauser C, et al A light-dependent molecular link between competition cues and defence responses in plants. Nat Plants. 2020:6(3):223–230. 10.1038/s41477-020-0604-8 32170284

[kiae045-B70] Feys B , Benedetti CE, Penfold CN, Turner JG. *Arabidopsis* mutants selected for resistance to the phytotoxin coronatine are male sterile, insensitive to methyl jasmonate, and resistant to a bacterial pathogen. Plant Cell. 1994:6(5):751–759. 10.2307/3869877 12244256 PMC160473

[kiae045-B71] Fonseca S , Chini A, Hamberg M, Adie B, Porzel A, Kramell R, Miersch O, Wasternack C, Solano R. (+)-7-iso-Jasmonoyl-L-isoleucine is the endogenous bioactive jasmonate. Nat Chem Biol. 2009:5(5):344–350. 10.1038/nchembio.161 19349968

[kiae045-B72] Fonseca S , Fernández-Calvo P, Fernández GM, Díez-Díaz M, Gimenez-Ibanez S, López-Vidriero I, Godoy M, Fernández-Barbero G, Van Leene J, De Jaeger G, et al bHLH003, bHLH013 and bHLH017 are new targets of JAZ repressors negatively regulating JA responses. PLoS One. 2014:9(1):e86182. 10.1371/journal.pone.0086182 24465948 PMC3900477

[kiae045-B73] Gao Y-Q , Jimenez-Sandoval P, Tiwari S, Stolz S, Wang J, Glauser G, Santiago J, Farmer EE. Ricca's factors as mobile proteinaceous effectors of electrical signaling. Cell. 2023:186(7):1337–1351.e20. 10.1016/j.cell.2023.02.006 36870332 PMC10098372

[kiae045-B74] Gasperini D , Chauvin A, Acosta IF, Kurenda A, Stolz S, Chételat A, Wolfender J-L, Farmer EE. Axial and radial oxylipin transport. Plant Physiol. 2015:169(3):2244–2254. 10.1104/pp.15.01104 26338953 PMC4634084

[kiae045-B75] Glauser G , Dubugnon L, Mousavi SAR, Rudaz S, Wolfender J-L, Farmer EE. Velocity estimates for signal propagation leading to systemic jasmonic acid accumulation in wounded *Arabidopsis*. J Biol Chem. 2009:284(50):34506–34513. 10.1074/jbc.M109.061432 19846562 PMC2787311

[kiae045-B76] Glazebrook J . Contrasting mechanisms of defense against biotrophic and necrotrophic pathogens. Annu Rev Phytopathol. 2005:43(1):205–227. 10.1146/annurev.phyto.43.040204.135923 16078883

[kiae045-B77] Goetz S , Hellwege A, Stenzel I, Kutter C, Hauptmann V, Forner S, McCaig B, Hause G, Miersch O, Wasternack C, et al Role of *cis*-12-oxo-phytodienoic acid in tomato embryo development. Plant Physiol. 2012:158(4):1715–1727. 10.1104/pp.111.192658 22337921 PMC3320180

[kiae045-B78] Grebner W , Stingl NE, Oenel A, Mueller MJ, Berger S. Lipoxygenase6-dependent oxylipin synthesis in roots is required for abiotic and biotic stress resistance of *Arabidopsis*. Plant Physiol. 2013:161(4):2159–2170. 10.1104/pp.113.214544 23444343 PMC3613484

[kiae045-B79] Green TR , Ryan CA. Wound-induced proteinase inhibitor in plant leaves: a possible defense mechanism against insects. Science. 1972:175(4023):776–777. 10.1126/science.175.4023.776 17836138

[kiae045-B80] Gundlach H , Müller MJ, Kutchan TM, Zenk MH. Jasmonic acid is a signal transducer in elicitor-induced plant cell cultures. Proc Natl Acad Sci U S A. 1992:89(6):2389–2393. 10.1073/pnas.89.6.2389 11607285 PMC48663

[kiae045-B81] Guo Q , Major IT, Kapali G, Howe GA. MYC transcription factors coordinate tryptophan-dependent defence responses and compromise seed yield in *Arabidopsis*. New Phytol. 2022:236(1):132–145. 10.1111/nph.18293 35642375 PMC9541860

[kiae045-B82] Guo Q , Yoshida Y, Major IT, Wang K, Sugimoto K, Kapali G, Havko NE, Benning C, Howe GA. JAZ repressors of metabolic defense promote growth and reproductive fitness in *Arabidopsis*. Proc Natl Acad Sci U S A. 2018:115(45):E10768–E10777. 10.1073/pnas.1811828115 30348775 PMC6233084

[kiae045-B83] He Z , Wang ZY, Li J, Zhu Q, Lamb C, Ronald P, Chory J. Perception of brassinosteroids by the extracellular domain of the receptor kinase BRI1. Science. 2000:288(5475):2360–2363. 10.1126/science.288.5475.2360 10875920

[kiae045-B84] Hedden P . The genes of the green revolution. Trends Genet. 2003:19(1):5–9. 10.1016/S0168-9525(02)00009-4 12493241

[kiae045-B85] Heitz T , Smirnova E, Widemann E, Aubert Y, Pinot F, Ménard R. The rise and fall of jasmonate biological activities. Subcell Biochem. 2016:86:405–426. 10.1007/978-3-319-25979-6_16 27023244

[kiae045-B86] Heitz T , Widemann E, Lugan R, Miesch L, Ullmann P, Désaubry L, Holder E, Grausem B, Kandel S, Miesch M, et al Cytochromes P450 CYP94C1 and CYP94B3 catalyze two successive oxidation steps of plant hormone jasmonoyl-isoleucine for catabolic turnover. J Biol Chem. 2012:287(9):6296–6306. 10.1074/jbc.M111.316364 22215670 PMC3307330

[kiae045-B87] Hilpert B , Bohlmann H, op den Camp RO, Przybyla D, Miersch O, Buchala A, Apel K. Isolation and characterization of signal transduction mutants of *Arabidopsis thaliana* that constitutively activate the octadecanoid pathway and form necrotic microlesions. Plant J. 2001:26(4):435–446. 10.1046/j.1365-313X.2001.2641036.x 11439130

[kiae045-B88] Howe GA . Plant hormones: metabolic end run to jasmonate. Nat Chem Biol. 2018:14(2):109–110. 10.1038/nchembio.2553 29337971

[kiae045-B89] Howe GA . Auspicious beginnings for the defense hormone jasmonate. Plant Cell. 2019:31(6):1198–1199. 10.1105/tpc.19.00333 31048334 PMC6588295

[kiae045-B90] Howe GA , Jander G. Plant immunity to insect herbivores. Annu Rev Plant Biol. 2008:59(1):41–66. 10.1146/annurev.arplant.59.032607.092825 18031220

[kiae045-B91] Howe GA , Major IT, Koo AJ. Modularity in jasmonate signaling for multistress resilience. Annu Rev Plant Biol. 2018:69(1):387–415. 10.1146/annurev-arplant-042817-040047 29539269

[kiae045-B92] Hu L . Integration of multiple volatile cues into plant defense responses. New Phytol. 2022:233(2):618–623. 10.1111/nph.17724 34506634

[kiae045-B93] Ishiguro S , Kawai-Oda A, Ueda J, Nishida I, Okada K. The *DEFECTIVE IN ANTHER DEHISCIENCE* gene encodes a novel phospholipase A1 catalyzing the initial step of jasmonic acid biosynthesis, which synchronizes pollen maturation, anther dehiscence, and flower opening in *Arabidopsis*. Plant Cell. 2001:13(10):2191–2209. 10.1105/tpc.010192 11595796 PMC139153

[kiae045-B94] Jensen AB , Raventos D, Mundy J. Fusion genetic analysis of jasmonate-signalling mutants in *Arabidopsis*. Plant J. 2002:29(5):595–606. 10.1046/j.0960-7412.2001.01241.x 11874572

[kiae045-B95] Jia K-P , Dickinson AJ, Mi J, Cui G, Xiao TT, Kharbatia NM, Guo X, Sugiono E, Aranda M, Blilou I, et al Anchorene is a carotenoid-derived regulatory metabolite required for anchor root formation in *Arabidopsis*. Sci Adv. 2019:5(11):eaaw6787. 10.1126/sciadv.aaw6787 31807696 PMC6881154

[kiae045-B96] Jung HW , Tschaplinski TJ, Wang L, Glazebrook J, Greenberg JT. Priming in systemic plant immunity. Science. 2009:324(5923):89–91. 10.1126/science.1170025 19342588

[kiae045-B97] Kagale S , Links MG, Rozwadowski K. Genome-wide analysis of ethylene-responsive element binding factor-associated amphiphilic repression motif-containing transcriptional regulators in *Arabidopsis*. Plant Physiol. 2010:152(3):1109–1134. 10.1104/pp.109.151704 20097792 PMC2832246

[kiae045-B98] Karban R , Baldwin IT. Induced responses to herbivory. Chicago: University of Chicago Press; 2007.

[kiae045-B99] Katsir L , Schilmiller AL, Staswick PE, He SY, Howe GA. COI1 is a critical component of a receptor for jasmonate and the bacterial virulence factor coronatine. Proc Natl Acad Sci U S A. 2008:105(19):7100–7105. 10.1073/pnas.0802332105 18458331 PMC2383947

[kiae045-B100] Katz E , Bagchi R, Jeschke V, Rasmussen ARM, Hopper A, Burow M, Estelle M, Kliebenstein DJ. Diverse allyl glucosinolate catabolites independently influence root growth and development. Plant Physiol. 2020:183(3):1376–1390. 10.1104/pp.20.00170 32321840 PMC7333702

[kiae045-B101] Katz E , Nisani S, Yadav BS, Woldemariam MG, Shai B, Obolski U, Ehrlich M, Shani E, Jander G, Chamovitz DA. The glucosinolate breakdown product indole-3-carbinol acts as an auxin antagonist in roots of *Arabidopsis thaliana*. Plant J. 2015:82(4):547–555. 10.1111/tpj.12824 25758811

[kiae045-B102] Kazan K , Manners JM. MYC2: the master in action. Mol Plant. 2013:6(3):686–703. 10.1093/mp/sss128 23142764

[kiae045-B103] Kende H . Plant biology and the Nobel Prize. Science. 1998:282(5389):627. 10.1126/science.282.5389.627b 9841411

[kiae045-B104] Kende H , Zeevaart J. The five “classical” plant hormones. Plant Cell. 1997:9:1197–1210. 10.1105/tpc.9.7.1197 12237383 PMC156991

[kiae045-B105] Kepinski S , Leyser O. The *Arabidopsis* F-box protein TIR1 is an auxin receptor. Nature. 2005:435(7041):446–451. 10.1038/nature03542 15917798

[kiae045-B106] Kim JI , Dolan WL, Anderson NA, Chapple C. Indole glucosinolate biosynthesis limits phenylpropanoid accumulation in *Arabidopsis thaliana*. Plant Cell. 2015:27(5):1529–1546. 10.1105/tpc.15.00127 25944103 PMC4456644

[kiae045-B107] Kneeshaw S , Soriano G, Monte I, Hamberg M, Zamarreño ÁM, García-Mina JM, Franco-Zorrilla JM, Kato N, Ueda M, Rey-Stolle MF, et al Ligand diversity contributes to the full activation of the jasmonate pathway in *Marchantia polymorpha*. Proc Natl Acad Sci U S A. 2022:119(36):e2202930119. 10.1073/pnas.2202930119 PMC9457472 36037336

[kiae045-B108] Knöfel H-D , Brückner C, Kramell R, Sembdner G, Schreiber K. A radioimmunoassay for jasmonic acid. Biochem Physiol Pflanz. 1984:179(4):317–325. 10.1016/S0015-3796(84)80048-7

[kiae045-B109] Koo AJ . Metabolism of the plant hormone jasmonate: a sentinel for tissue damage and master regulator of stress response. Phytochem Rev. 2018:17(1):51–80. 10.1007/s11101-017-9510-8

[kiae045-B110] Koo AJK , Cooke TF, Howe GA. Cytochrome P450 CYP94B3 mediates catabolism and inactivation of the plant hormone jasmonoyl-L-isoleucine. Proc Natl Acad Sci U S A. 2011:108(22):9298–9303. 10.1073/pnas.1103542108 21576464 PMC3107288

[kiae045-B111] Koo AJK , Gao X, Jones AD, Howe GA. A rapid wound signal activates the systemic synthesis of bioactive jasmonates in *Arabidopsis*. Plant J. 2009:59(6):974–986. 10.1111/j.1365-313X.2009.03924.x 19473329

[kiae045-B112] Koo AJK , Howe GA. The wound hormone jasmonate. Phytochemistry. 2009:70(13–14):1571–1580. 10.1016/j.phytochem.2009.07.018 19695649 PMC2784233

[kiae045-B113] Koo AJ , Thireault C, Zemelis S, Poudel AN, Zhang T, Kitaoka N, Brandizzi F, Matsuura H, Howe GA. Endoplasmic reticulum-associated inactivation of the hormone jasmonoyl-L-isoleucine by multiple members of the cytochrome P450 94 family in *Arabidopsis*. J Biol Chem. 2014:289(43):29728–29738. 10.1074/jbc.M114.603084 25210037 PMC4207986

[kiae045-B114] Laha D , Johnen P, Azevedo C, Dynowski M, Weiß M, Capolicchio S, Mao H, Iven T, Steenbergen M, Freyer M, et al VIH2 regulates the synthesis of inositol pyrophosphate InsP_8_ and jasmonate-dependent defenses in *Arabidopsis*. Plant Cell. 2015:27(4):1082–1097. 10.1105/tpc.114.135160 25901085 PMC4558690

[kiae045-B115] Lavecchia A . Machine-learning approaches in drug discovery: methods and applications. Drug Discov Today. 2015:20(3):318–331. 10.1016/j.drudis.2014.10.012 25448759

[kiae045-B116] Li L-L , Li Z, Lou Y, Meiners SJ, Kong C-H. (−)-Loliolide is a general signal of plant stress that activates jasmonate-related responses. New Phytol. 2022:238(5):2099–2112. 10.1111/nph.18644 36444519

[kiae045-B117] Li M , Wang F, Li S, Yu G, Wang L, Li Q, Zhu X, Li Z, Yuan L, Liu P. Importers drive leaf-to-leaf jasmonic acid transmission in wound-induced systemic immunity. Mol Plant. 2020:13(10):1485–1498. 10.1016/j.molp.2020.08.017 32889174

[kiae045-B118] Li M , Yu G, Cao C, Liu P. Metabolism, signaling, and transport of jasmonates. Plant Commun. 2021:2(5):100231. 10.1016/j.xplc.2021.100231 34746762 PMC8555440

[kiae045-B119] Li L , Zhao Y, McCaig BC, Wingerd BA, Wang J, Whalon ME, Pichersky E, Howe GA. The tomato homolog of CORONATINE-INSENSITIVE1 is required for the maternal control of seed maturation, jasmonate-signaled defense responses, and glandular trichome development. Plant Cell. 2004:16(1):126–143. 10.1105/tpc.017954 14688297 PMC301400

[kiae045-B120] Liu K-H , Liu M, Lin Z, Wang Z-F, Chen B, Liu C, Guo A, Konishi M, Yanagisawa S, Wagner G, et al NIN-like protein 7 transcription factor is a plant nitrate sensor. Science. 2022:377(6613):1419–1425. 10.1126/science.add1104 36137053 PMC9628810

[kiae045-B121] Lorenzo O , Chico JM, Sánchez-Serrano JJ, Solano R. *JASMONATE-INSENSITIVE1* encodes a MYC transcription factor essential to discriminate between different jasmonate-regulated defense responses in *Arabidopsis*. Plant Cell. 2004:16(7):1938–1950. 10.1105/tpc.022319 15208388 PMC514172

[kiae045-B122] Luna E , van Hulten M, Zhang Y, Berkowitz O, López A, Pétriacq P, Sellwood MA, Chen B, Burrell M, van de Meene A, et al Plant perception of β-aminobutyric acid is mediated by an aspartyl-tRNA synthetase. Nat Chem Biol. 2014:10(6):450–456. 10.1038/nchembio.1520 24776930 PMC4028204

[kiae045-B123] Luzarowski M , Skirycz A. Emerging strategies for the identification of protein–metabolite interactions. J Exp Bot. 2019:70(18):4605–4618. 10.1093/jxb/erz228 31087097 PMC6760282

[kiae045-B124] Maeda HA , Fernie AR. Evolutionary history of plant metabolism. Annu Rev Plant Biol. 2021:72(1):185–216. 10.1146/annurev-arplant-080620-031054 33848429

[kiae045-B125] Malinovsky FG , Thomsen M-LF, Nintemann SJ, Jagd LM, Bourgine B, Burow M, Kliebenstein DJ. An evolutionarily young defense metabolite influences the root growth of plants via the ancient TOR signaling pathway. eLife. 2017:6:e29353. 10.7554/eLife.29353 29231169 PMC5730369

[kiae045-B126] Matsui K . Green leaf volatiles: hydroperoxide lyase pathway of oxylipin metabolism. Curr Opin Plant Biol. 2006:9(3):274–280. 10.1016/j.pbi.2006.03.002 16595187

[kiae045-B127] McConn M , Browse J. The critical requirement for linolenic acid is pollen development, not photosynthesis, in an *Arabidopsis* mutant. Plant Cell. 1996:8(3):403–416. 10.2307/3870321 12239389 PMC161109

[kiae045-B128] Melotto M , Mecey C, Niu Y, Chung HS, Katsir L, Yao J, Zeng W, Thines B, Staswick P, Browse J, et al A critical role of two positively charged amino acids in the jas motif of *Arabidopsis* JAZ proteins in mediating coronatine- and jasmonoyl isoleucine-dependent interactions with the COI1 F-box protein. Plant J. 2008:55(6):979–988. 10.1111/j.1365-313X.2008.03566.x 18547396 PMC2653208

[kiae045-B129] Meyerowitz EM . Plants compared to animals: the broadest comparative study of development. Science. 2002:295(5559):1482–1485. 10.1126/science.1066609 11859185

[kiae045-B130] Mochizuki S , Sugimoto K, Koeduka T, Matsui K. *Arabidopsis* lipoxygenase 2 is essential for formation of green leaf volatiles and five-carbon volatiles. FEBS Lett. 2016:590(7):1017–1027. 10.1002/1873-3468.12133 26991128

[kiae045-B131] Monte I . Jasmonates and salicylic acid: evolution of defense hormones in land plants. Curr Opin Plant Biol. 2023:76:102470. 10.1016/j.pbi.2023.102470 37801737

[kiae045-B132] Monte I , Franco-Zorrilla JM, García-Casado G, Zamarreño AM, García-Mina JM, Nishihama R, Kohchi T, Solano R. A single JAZ repressor controls the jasmonate pathway in *Marchantia* polymorpha. Mol Plant. 2019:12(2):185–198. 10.1016/j.molp.2018.12.017 30594656

[kiae045-B133] Monte I , Ishida S, Zamarreño AM, Hamberg M, Franco-Zorrilla JM, García-Casado G, Gouhier-Darimont C, Reymond P, Takahashi K, García-Mina JM, et al Ligand–receptor co-evolution shaped the jasmonate pathway in land plants. Nat Chem Biol. 2018:14(5):480–488. 10.1038/s41589-018-0033-4 29632411

[kiae045-B134] Monte I , Kneeshaw S, Franco-Zorrilla JM, Chini A, Zamarreño AM, García-Mina JM, Solano R. An ancient COI1-independent function for reactive electrophilic oxylipins in thermotolerance. Curr Biol. 2020:30(6):962–971.e3. 10.1016/j.cub.2020.01.023 32142692

[kiae045-B135] Moreno JE , Shyu C, Campos ML, Patel LC, Chung HS, Yao J, He SY, Howe GA. Negative feedback control of jasmonate signaling by an alternative splice variant of JAZ10. Plant Physiol. 2013:162(2):1006–1017. 10.1104/pp.113.218164 23632853 PMC3668036

[kiae045-B136] Morffy N , Strader LC. Old town roads: routes of auxin biosynthesis across kingdoms. Curr Opin Plant Biol. 2020:55:21–27. 10.1016/j.pbi.2020.02.002 32199307 PMC7540728

[kiae045-B137] Morin H , Chételat A, Stolz S, Marcourt L, Glauser G, Wolfender J-L, Farmer EE. Wound-response jasmonate dynamics in the primary vasculature. New Phytol. 2023:240(4):1484–1496. 10.1111/nph.19207 37598308

[kiae045-B138] Mousavi SAR , Chauvin A, Pascaud F, Kellenberger S, Farmer EE. GLUTAMATE RECEPTOR-LIKE genes mediate leaf-to-leaf wound signalling. Nature. 2013:500(7463):422–426. 10.1038/nature12478 23969459

[kiae045-B139] Müller A , Düchting P, Weiler EW. A multiplex GC-MS/MS technique for the sensitive and quantitative single-run analysis of acidic phytohormones and related compounds, and its application to *Arabidopsis thaliana*. Planta. 2002:216(1):44–56. 10.1007/s00425-002-0866-6 12430013

[kiae045-B140] Mutte SK , Kato H, Rothfels C, Melkonian M, Wong GK-S, Weijers D. Origin and evolution of the nuclear auxin response system. eLife. 2018:7:e33399. 10.7554/eLife.33399 29580381 PMC5873896

[kiae045-B141] Nagashima A , Higaki T, Koeduka T, Ishigami K, Hosokawa S, Watanabe H, Matsui K, Hasezawa S, Touhara K. Transcriptional regulators involved in responses to volatile organic compounds in plants. J Biol Chem. 2019:294(7):2256–2266. 10.1074/jbc.RA118.005843 30593507 PMC6378981

[kiae045-B142] Nakata M , Mitsuda N, Herde M, Koo AJK, Moreno JE, Suzuki K, Howe GA, Ohme-Takagi M. A bHLH-type transcription factor, ABA-INDUCIBLE BHLH-TYPE TRANSCRIPTION FACTOR/JA-ASSOCIATED MYC2-LIKE1, acts as a repressor to negatively regulate jasmonate signaling in *Arabidopsis*. Plant Cell. 2013:25(5):1641–1656. 10.1105/tpc.113.111112 23673982 PMC3694697

[kiae045-B143] Nguyen CT , Kurenda A, Stolz S, Chételat A, Farmer EE. Identification of cell populations necessary for leaf-to-leaf electrical signaling in a wounded plant. Proc Natl Acad Sci U S A. 2018:115(40):10178–10183. 10.1073/pnas.1807049115 30228123 PMC6176584

[kiae045-B144] Ninkovic V , Markovic D, Rensing M. Plant volatiles as cues and signals in plant communication. Plant Cell Environ. 2021:44(4):1030–1043. 10.1111/pce.13910 33047347 PMC8048923

[kiae045-B145] Parthier B . Jasmonates: hormonal regulators or stress factors in leaf senescence? J Plant Growth Regul. 1990:9(1–4):57. 10.1007/BF02041942

[kiae045-B146] Pauwels L , Barbero GF, Geerinck J, Tilleman S, Grunewald W, Pérez AC, Chico JM, Bossche RV, Sewell J, Gil E, et al NINJA connects the co-repressor TOPLESS to jasmonate signalling. Nature. 2010:464(7289):788–791. 10.1038/nature08854 20360743 PMC2849182

[kiae045-B147] Peer WA , Murphy AS. Flavonoids and auxin transport: modulators or regulators? Trends Plant Sci. 2007:12(12):556–563. 10.1016/j.tplants.2007.10.003 18198522

[kiae045-B148] Peñuelas M , Monte I, Schweizer F, Vallat A, Reymond P, García-Casado G, Franco-Zorrilla JM, Solano R. Jasmonate-related MYC transcription factors are functionally conserved in *Marchantia polymorpha*. Plant Cell. 2019:31(10):2491–2509. 10.1105/tpc.18.00974 31391256 PMC6790078

[kiae045-B149] Piazza I , Kochanowski K, Cappelletti V, Fuhrer T, Noor E, Sauer U, Picotti P. A map of protein-metabolite interactions reveals principles of chemical communication. Cell. 2018:172(1–2):358–372.e23. 10.1016/j.cell.2017.12.006 29307493

[kiae045-B150] Pourcel L , Irani NG, Koo AJK, Bohorquez-Restrepo A, Howe GA, Grotewold E. A chemical complementation approach reveals genes and interactions of flavonoids with other pathways. Plant J. 2013:74(3):383–397. 10.1111/tpj.12129 23360095

[kiae045-B151] Qi L , Kwiatkowski M, Chen H, Hoermayer L, Sinclair S, Zou M, Del Genio CI, Kubeš MF, Napier R, Jaworski K, et al Adenylate cyclase activity of TIR1/AFB auxin receptors in plants. Nature. 2022:611(7934):133–138. 10.1038/s41586-022-05369-7 36289340

[kiae045-B152] Qi T , Wang J, Huang H, Liu B, Gao H, Liu Y, Song S, Xie D. Regulation of jasmonate-induced leaf senescence by antagonism between bHLH subgroup IIIe and IIId factors in *Arabidopsis*. Plant Cell. 2015:27(6):1634–1649. 10.1105/tpc.15.00110 26071420 PMC4498205

[kiae045-B153] Ray R , Halitschke R, Gase K, Leddy SM, Schuman MC, Rodde N, Baldwin IT. A persistent major mutation in canonical jasmonate signaling is embedded in an herbivory-elicited gene network. Proc Natl Acad Sci U S A. 2023:120(35):e2308500120. 10.1073/pnas.2308500120 PMC10466192 37607232

[kiae045-B154] Reymond P , Weber H, Damond M, Farmer EE. Differential gene expression in response to mechanical wounding and insect feeding in Arabidopsis. Plant Cell. 2000:12:707–720. 10810145 10.1105/tpc.12.5.707 PMC139922

[kiae045-B156] Rieseberg TP , Dadras A, Fürst-Jansen JMR, Dhabalia Ashok A, Darienko T, de Vries S, Irisarri I, de Vries J. Crossroads in the evolution of plant specialized metabolism. Semin Cell Dev Biol. 2023:134:37–58. 10.1016/j.semcdb.2022.03.004 35292191

[kiae045-B157] Robert-Seilaniantz A , Grant M, Jones JDG. Hormone crosstalk in plant disease and defense: more than just jasmonate-salicylate antagonism. Annu Rev Phytopathol. 2011:49(1):317–343. 10.1146/annurev-phyto-073009-114447 21663438

[kiae045-B158] Ruegger M , Dewey E, Gray WM, Hobbie L, Turner J, Estelle M. The TIR1 protein of *Arabidopsis* functions in auxin response and is related to human SKP2 and yeast grr1p. Genes Dev. 1998:12(2):198–207. 10.1101/gad.12.2.198 9436980 PMC316440

[kiae045-B159] Salehin M , Li B, Tang M, Katz E, Song L, Ecker JR, Kliebenstein DJ, Estelle M. Auxin-sensitive aux/IAA proteins mediate drought tolerance in Arabidopsis by regulating glucosinolate levels. Nat Commun. 2019:10(1):4021. 10.1038/s41467-019-12002-1 31492889 PMC6731224

[kiae045-B160] Sandoval PJ , Santiago J. In vitro analytical approaches to study plant ligand-receptor interactions. Plant Physiol. 2020:182(4):1697–1712. 10.1104/pp.19.01396 32034053 PMC7140929

[kiae045-B161] Santner A , Calderon-Villalobos LIA, Estelle M. Plant hormones are versatile chemical regulators of plant growth. Nat Chem Biol. 2009:5(5):301–307. 10.1038/nchembio.165 19377456

[kiae045-B162] Schaller F , Weiler EW. Enzymes of octadecanoid biosynthesis in plants–12-oxo-phytodienoate 10,11-reductase. Eur J Biochem. 1997:245(2):294–299. 10.1111/j.1432-1033.1997.t01-1-00294.x 9151956

[kiae045-B163] Schilmiller AL , Koo AJK, Howe GA. Functional diversification of acyl-coenzyme A oxidases in jasmonic acid biosynthesis and action. Plant Physiol. 2007:143(2):812–824. 10.1104/pp.106.092916 17172287 PMC1803733

[kiae045-B164] Schlossarek D , Zhang Y, Sokolowska EM, Fernie AR, Luzarowski M, Skirycz A. Don’t let go: co-fractionation mass spectrometry for untargeted mapping of protein-metabolite interactomes. Plant J. 2023:113(5):904–914. 10.1111/tpj.16084 36575913

[kiae045-B165] Schneeberger K , Ossowski S, Lanz C, Juul T, Petersen AH, Nielsen KL, Jørgensen J-E, Weigel D, Andersen SU. SHOREmap: simultaneous mapping and mutation identification by deep sequencing. Nat Methods. 2009:6(8):550–551. 10.1038/nmeth0809-550 19644454

[kiae045-B166] Schulze A , Zimmer M, Mielke S, Stellmach H, Melnyk CW, Hause B, Gasperini D. Wound-Induced shoot-to-root relocation of JA-Ile precursors coordinates *Arabidopsis* growth. Mol Plant. 2019:12(10):1383–1394. 10.1016/j.molp.2019.05.013 31181337

[kiae045-B167] Sembdner G , Parthier B. The biochemistry and the physiological and molecular actions of jasmonates. Annu Rev Plant Physiol Plant Mol Biol. 1993:44(1):569–589. 10.1146/annurev.pp.44.060193.003033

[kiae045-B168] Shabek N , Zheng N. Plant ubiquitin ligases as signaling hubs. Nat Struct Mol Biol. 2014:21(4):293–296. 10.1038/nsmb.2804 24699076

[kiae045-B169] Sheard LB , Tan X, Mao H, Withers J, Ben-Nissan G. Jasmonate perception by inositol-phosphate-potentiated COI1–JAZ co-receptor. Nature. 2010:468(7322):400–405. 10.1038/nature09430 20927106 PMC2988090

[kiae045-B170] Shields A , Shivnauth V, Castroverde CDM. Salicylic acid and *N*-hydroxypipecolic acid at the fulcrum of the plant immunity-growth equilibrium. Front Plant Sci. 2022:13:841688. 10.3389/fpls.2022.841688 35360332 PMC8960316

[kiae045-B171] Shyu C , Figueroa P, Depew CL, Cooke TF, Sheard LB, Moreno JE, Katsir L, Zheng N, Browse J, Howe GA. JAZ8 lacks a canonical degron and has an EAR motif that mediates transcriptional repression of jasmonate responses in *Arabidopsis*. Plant Cell. 2012:24(2):536–550. 10.1105/tpc.111.093005 22327740 PMC3315231

[kiae045-B172] Smirnova E , Marquis V, Poirier L, Aubert Y, Zumsteg J, Ménard R, Miesch L, Heitz T. Jasmonic acid oxidase 2 hydroxylates jasmonic acid and represses basal defense and resistance responses against *Botrytis cinerea* infection. Mol Plant. 2017:10(9):1159–1173. 10.1016/j.molp.2017.07.010 28760569

[kiae045-B173] Song WC , Brash AR. Purification of an allene oxide synthase and identification of the enzyme as a cytochrome P-450. Science. 1991:253(5021):781–784. 10.1126/science.1876834 1876834

[kiae045-B174] Song S , Qi T, Wasternack C, Xie D. Jasmonate signaling and crosstalk with gibberellin and ethylene. Curr Opin Plant Biol. 2014:21:112–119. 10.1016/j.pbi.2014.07.005 25064075

[kiae045-B175] Staswick PE . Novel regulation of vegetative storage protein genes. Plant Cell. 1990:2(1):1–6. 10.2307/3869045 12354941 PMC159858

[kiae045-B176] Staswick PE , Su W, Howell SH. Methyl jasmonate inhibition of root growth and induction of a leaf protein are decreased in an *Arabidopsis thaliana* mutant. Proc Natl Acad Sci U S A. 1992:89(15):6837–6840. 10.1073/pnas.89.15.6837 11607311 PMC49599

[kiae045-B177] Staswick PE , Tiryaki I. The oxylipin signal jasmonic acid is activated by an enzyme that conjugates it to isoleucine in *Arabidopsis*. Plant Cell. 2004:16(8):2117–2127. 10.1105/tpc.104.023549 15258265 PMC519202

[kiae045-B178] Staswick PE , Tiryaki I, Rowe ML. Jasmonate response locus *JAR1* and several related *Arabidopsis* genes encode enzymes of the firefly luciferase superfamily that show activity on jasmonic, salicylic, and indole-3-acetic acids in an assay for adenylation. Plant Cell. 2002:14(6):1405–1415. 10.1105/tpc.000885 12084835 PMC150788

[kiae045-B179] Stumpe M , Göbel C, Faltin B, Beike AK, Hause B, Himmelsbach K, Bode J, Kramell R, Wasternack C, Frank W, et al The moss *Physcomitrella patens* contains cyclopentenones but no jasmonates: mutations in allene oxide cyclase lead to reduced fertility and altered sporophyte morphology. New Phytol. 2010:188(3):740–749. 10.1111/j.1469-8137.2010.03406.x 20704658

[kiae045-B180] Sugimoto K , Zager JJ, Aubin BS, Lange BM, Howe GA. Flavonoid deficiency disrupts redox homeostasis and terpenoid biosynthesis in glandular trichomes of tomato. Plant Physiol. 2022:188(3):1450–1468. 10.1093/plphys/kiab488 34668550 PMC8896623

[kiae045-B181] Sun Y , Harpazi B, Wijerathna-Yapa A, Merilo E, de Vries J, Michaeli D, Gal M, Cuming AC, Kollist H, Mosquna A. A ligand-independent origin of abscisic acid perception. Proc Natl Acad Sci U S A. 2019:116(49):24892–24899. 10.1073/pnas.1914480116 31744875 PMC6900503

[kiae045-B182] Takeuchi J , Fukui K, Seto Y, Takaoka Y, Okamoto M. Ligand-receptor interactions in plant hormone signaling. Plant J. 2021:105(2):290–306. 10.1111/tpj.15115 33278046

[kiae045-B183] Tan X , Calderon-Villalobos LIA, Sharon M, Zheng C, Robinson CV, Estelle M, Zheng N. Mechanism of auxin perception by the TIR1 ubiquitin ligase. Nature. 2007:446(7136):640–645. 10.1038/nature05731 17410169

[kiae045-B184] Tata JR . One hundred years of hormones. EMBO Rep. 2005:6(6):490–496. 10.1038/sj.embor.7400444 15940278 PMC1369102

[kiae045-B185] Thines B , Katsir L, Melotto M, Niu Y, Mandaokar A, Liu G, Nomura K, He SY, Howe GA, Browse J. JAZ repressor proteins are targets of the SCF(COI1) complex during jasmonate signalling. Nature. 2007:448(7154):661–665. 10.1038/nature05960 17637677

[kiae045-B186] Thireault C , Shyu C, Yoshida Y, St Aubin B, Campos ML, Howe GA. Repression of jasmonate signaling by a non-TIFY JAZ protein in *Arabidopsis*. Plant J. 2015:82(4):669–679. 10.1111/tpj.12841 25846245

[kiae045-B187] Thomma BP , Eggermont K, Penninckx IA, Mauch-Mani B, Vogelsang R, Cammue BP, Broekaert WF. Separate jasmonate-dependent and salicylate-dependent defense-response pathways in *Arabidopsis* are essential for resistance to distinct microbial pathogens. Proc Natl Acad Sci U S A. 1998:95(25):15107–15111. 10.1073/pnas.95.25.15107 9844023 PMC24583

[kiae045-B188] Toyota M , Spencer D, Sawai-Toyota S, Jiaqi W, Zhang T, Koo AJ, Howe GA, Gilroy S. Glutamate triggers long-distance, calcium-based plant defense signaling. Science. 2018:361(6407):1112–1115. 10.1126/science.aat7744 30213912

[kiae045-B189] Trewavas A . How do plant growth substances work? Plant Cell Environ. 1981:4(3):203–228. 10.1111/j.1365-3040.1981.tb01048.x

[kiae045-B190] Ueda J , Kato J. Isolation and identification of a senescence-promoting substance from wormwood (*Artemisia absinthium* L). Plant Physiol. 1980:66(2):246–249. 10.1104/pp.66.2.246 16661414 PMC440575

[kiae045-B191] Urano D , Jones AM. Heterotrimeric G protein-coupled signaling in plants. Annu Rev Plant Biol. 2014:65(1):365–384. 10.1146/annurev-arplant-050213-040133 24313842 PMC4861148

[kiae045-B192] Vick BA , Zimmerman DC. Biosynthesis of jasmonic acid by several plant species. Plant Physiol. 1984:75(2):458–461. 10.1104/pp.75.2.458 16663643 PMC1066929

[kiae045-B193] Wang K-D , Borrego EJ, Kenerley CM, Kolomeits MV. Oxylipins other than jasmonic acid are xylem-resident signals regulating systemic resistance induced by *Trichoderma virens* in maize. Plant Cell. 2020:32(1):16–185. 10.1105/tpc.19.00487 PMC6961617 31690653

[kiae045-B194] Wang L , Erb M. Volatile uptake, transport, perception, and signaling shape a plant's nose. Essays Biochem. 2022:66(5):695–702. 10.1042/EBC20210092 36062590 PMC9528081

[kiae045-B195] Wang JY , Haider I, Jamil M, Fiorilli V, Saito Y, Mi J, Baz L, Kountche BA, Jia K-P, Guo X, et al The apocarotenoid metabolite zaxinone regulates growth and strigolactone biosynthesis in rice. Nat Commun. 2019:10(1):810. 10.1038/s41467-019-08461-1 30778050 PMC6379432

[kiae045-B196] Wang JY , Lin P-Y, Al-Babili S. On the biosynthesis and evolution of apocarotenoid plant growth regulators. Semin Cell Dev Biol. 2021:109:3–11. 10.1016/j.semcdb.2020.07.007 32732130

[kiae045-B197] Wang J , Wu D, Wang Y, Xie D. Jasmonate action in plant defense against insects. J Exp Bot. 2019:70(13):3391–3400. 10.1093/jxb/erz174 30976791

[kiae045-B198] Wasternack C . How jasmonates earned their laurels: past and present. J Plant Growth Regul. 2015:34(4):761–794. 10.1007/s00344-015-9526-5

[kiae045-B199] Wasternack C , Feussner I. The oxylipin pathways: biochemistry and function. Annu Rev Plant Biol. 2018:69(1):363–386. 10.1146/annurev-arplant-042817-040440 29166128

[kiae045-B200] Wasternack C , Parthier B. Jasmonate-signalled plant gene expression. Trends Plant Sci. 1997:2(8):302–307. 10.1016/S1360-1385(97)89952-9

[kiae045-B201] Wasternack C , Song S. Jasmonates: biosynthesis, metabolism, and signaling by proteins activating and repressing transciption. J Exp Bot. 2017:68(6)1303–1321. 10.1093/jxb/erw443 27940470

[kiae045-B202] Waters MT , Nelson DC. Karrikin perception and signalling. New Phytol. 2023:237(3):1525–1541. 10.1111/nph.18598 36333982

[kiae045-B203] Weng J-K , Lynch JH, Matos JO, Dudareva N. Adaptive mechanisms of plant specialized metabolism connecting chemistry to function. Nat Chem Biol. 2021:17(10):1037–1045. 10.1038/s41589-021-00822-6 34552220

[kiae045-B204] Westfall CS , Zubieta C, Herrmann J, Kapp U, Nanao MH, Jez JM. Structural basis for prereceptor modulation of plant hormones by GH3 proteins. Science. 2012:336(6089):1708–1711. 10.1126/science.1221863 22628555

[kiae045-B205] Widemann E , Miesch L, Lugan R, Holder E, Heinrich C, Aubert Y, Miesch M, Pinot F, Heitz T. The amidohydrolases IAR3 and ILL6 contribute to jasmonoyl-isoleucine hormone turnover and generate 12-hydroxyjasmonic acid upon wounding in *Arabidopsis* leaves. J Biol Chem. 2013:288(44):31701–31714. 10.1074/jbc.M113.499228 24052260 PMC3814765

[kiae045-B206] Woldemariam MG , Onkokesung N, Baldwin IT, Galis I. Jasmonoyl-L-isoleucine hydrolase 1 (JIH1) regulates jasmonoyl-L-isoleucine levels and attenuates plant defenses against herbivores. Plant J. 2012:72(5):758–767. 10.1111/j.1365-313X.2012.05117.x 22860609

[kiae045-B207] Wong A , Chi W, Yu J, Bi C, Tian X, Yang Y, Gehring C. Plant adenylate cyclases have come full circle. Nat Plants. 2023:9(9):1389–1397. 10.1038/s41477-023-01486-x 37709954

[kiae045-B208] Wu F , Deng L, Zhai Q, Zhao J, Chen Q, Li C. Mediator subunit MED25 couples alternative splicing of *JAZ* genes with fine-tuning of jasmonate signaling. Plant Cell. 2020:32(2):429–448. 10.1105/tpc.19.00583 31852773 PMC7008490

[kiae045-B209] Xie DX , Feys BF, James S, Nieto-Rostro M, Turner JG. *COI1*: an *Arabidopsis* gene required for jasmonate-regulated defense and fertility. Science. 1998:280(5366):1091–1094. 10.1126/science.280.5366.1091 9582125

[kiae045-B210] Xu L , Liu F, Wang Z, Peng W, Huang R, Huang D, Xie D. An *Arabidopsis* mutant *cex1* exhibits constant accumulation of jasmonate-regulated *AtVSP*, *Thi2.1* and *PDF1.2*. FEBS Lett. 2001:494(3):161–164. 10.1016/S0014-5793(01)02331-6 11311233

[kiae045-B211] Yamane H , Takahashi N, Ueda J-I, Kato J. Resolution of (±)-methyl jasmonate by high performance liquid chromatography and the inhibitory effect of (+)-enantiomer on the growth of rice seedlings. Agric Biol Chem. 1981:45(7):1709–1711. 10.1080/00021369.1981.10864755

[kiae045-B212] Yan Y , Stolz S, Chételat A, Reymond P, Pagni M, Dubugnon L, Farmer EE. A downstream mediator in the growth repression limb of the jasmonate pathway. Plant Cell. 2007:19(8):2470–2483. 10.1105/tpc.107.050708 17675405 PMC2002611

[kiae045-B213] Yan J , Yao R, Chen L, Li S, Gu M, Nan F, Xie D. Dynamic perception of jasmonates by the F-box protein COI1. Mol Plant. 2018:11:1237–1247. 10.1016/j.molp.2018.07.007 30092285

[kiae045-B214] Yu Y , Tang W, Lin W, Li W, Zhou X, Li Y, Chen R, Zheng R, Qin G, Cao W, et al ABLs and TMKs are co-receptors for extracellular auxin. Cell. 2023:186(25):5457–5471.e17. 10.1016/j.cell.2023.10.017 37979582 PMC10827329

[kiae045-B215] Zander M . Many ways to repress! JAZ's agony of choices. Mol Plant. 2021:14(5):714–716. 10.1016/j.molp.2021.04.010 33872770

[kiae045-B216] Zander M , Lewsey MG, Clark NM, Yin L, Bartlett A, Saldierna Guzmán JP, Hann E, Langford AE, Jow B, Wise A, et al Integrated multi-omics framework of the plant response to jasmonic acid. Nat Plants. 2020:6(3):290–302. 10.1038/s41477-020-0605-7 32170290 PMC7094030

[kiae045-B217] Zhang F , Ke J, Zhang L, Chen R, Sugimoto K, Howe GA, Xu HE, Zhou M, He SY, Melcher K. Structural insights into alternative splicing-mediated desensitization of jasmonate signaling. Proc Natl Acad Sci U S A. 2017:114(7):1720–1725. 10.1073/pnas.1616938114 28137867 PMC5320967

[kiae045-B218] Zhang Y , Turner JG. Wound-induced endogenous jasmonates stunt plant growth by inhibiting mitosis. PLoS One. 2008:3(11):e3699. 10.1371/journal.pone.0003699 19002244 PMC2577035

[kiae045-B219] Zhang F , Yao J, Ke J, Zhang L, Lam VQ, Xin X-F, Zhou XE, Chen J, Brunzelle J, Griffin PR, et al Structural basis of JAZ repression of MYC transcription factors in jasmonate signalling. Nature. 2015:525(7568):269–273. 10.1038/nature14661 26258305 PMC4567411

[kiae045-B220] Zhou XE , Zhang Y, Yao J, Zheng J, Zhou Y, He Q, Moreno J, Lam VQ, Cao X, Sugimoto K, et al Assembly of JAZ-JAZ and JAZ-NINJA complexes in jasmonate signaling. Plant Commun. 2023:4(6):100639–. 10.1016/j.xplc.2023.100639 37322867 PMC10721472

[kiae045-B221] Zuo Z , Weraduwage SM, Lantz AT, Sanchez LM, Weise SE, Wang J, Childs KL, Sharkey TD. Isoprene acts as a signaling molecule in gene networks important for stress responses and plant growth. Plant Physiol. 2019:180(1):124–152. 10.1104/pp.18.01391 30760638 PMC6501071

